# Biochemical and Molecular Fingerprinting of Newly Developed *Phaseolus vulgaris* L. Genotypes: Unveiling Superior Germplasm for Food Security

**DOI:** 10.3390/foods15193576

**Published:** 2026-10-08

**Authors:** Aurora Dobrin, Liliana Bădulescu, Carmen-Gabriela Constantin, Violeta Alexandra Ion, Oana Crina Bujor-Nenița, Andrei Cătălin Petre, Mihai Frîncu, Anca Amalia Udriște, Maria Ojog, Bianca Mușat

**Affiliations:** 1 Research Center for Studies of Food Quality and Agricultural Products, University of Agronomic Sciences and Veterinary Medicine of Bucharest, 59 Mărăști Blvd, 011464 Bucharest, Romania; aurora.dobrin@qlab.usamv.ro (A.D.); liliana.badulescu@qlab.usamv.ro (L.B.); violeta.ion@qlab.usamv.ro (V.A.I.); oana.bujor@qlab.usamv.ro (O.C.B.-N.); andrei.petre@qlab.usamv.ro (A.C.P.); mihai.frincu@qlab.usamv.ro (M.F.); amalia.udriste@qlab.usamv.ro (A.A.U.); 2 Faculty of Horticulture, University of Agronomic Sciences and Veterinary Medicine of Bucharest, 59 Mărăști Blvd, 011464 Bucharest, Romania; maria.ojog@horti.usamv.ro; 3 Plant Genetic Resources Bank-for Vegetables, Floricultural, Aromatic and Medicinal Plants, 56 Nicolae Bălcescu Bldv, 120246 Buzău, Romania; zamfir_b@yahoo.com

**Keywords:** nutritional, bean pods, antioxidant capacity, minerals, protein, ISSR markers

## Abstract

The common bean (*Phaseolus vulgaris* L.) is a sustainable source of plant protein, dietary fiber, polyphenols, essential minerals, and antioxidant pigments, increasingly valued as a raw material for functional foods and nutraceutical ingredients. Consistent compositional characterization of new lines is therefore essential both for nutritional labeling and authenticating food-grade germplasm. This study characterizes the nutritional profile, bioactive compounds, antioxidant activity, and mineral contribution of three new bean genotypes grown in Buzău County, Romania, across two consecutive harvest years, complemented by ISSR molecular fingerprinting for cultivar authentication. Genotypes were evaluated for dry matter, total soluble solids, titratable acidity, assimilatory pigments, total phenolic content, total flavonoid content, antioxidant capacity, crude fiber, total sugars, protein, starch, ash, and microelements (Mn, Cu, Zn) and macroelements (Na, Mg, K, Ca) with nutrient reference values (NRVs) per 100 g. Distinct compositional profiles supported differentiated food applications: L BEC (green, long pod) recorded the highest protein, soluble sugars, titratable acidity, pigments, total polyphenols, antioxidant capacity, Na, Mg, Mn, and Zn, indicating suitability for functional and nutraceutical formulations. L 301B (purple pod) accumulated the highest dry matter, ash, crude fiber, total flavonoids, K, and Ca, favoring high-fiber and mineral-fortified products. L HELD (green, flattened pod) yielded the highest °Brix, starch, and Cu, suited to starch-rich, energy-dense foods. All genotypes delivered nutritionally significant amounts of Mg, K, Ca, Mn, Cu, and Zn under Regulation (EU) No 1169/2011. ISSR analysis revealed clear polymorphism and genotype-specific banding patterns, and inter-annual compositional consistency indicated the reliability of these lines as standardized food raw materials. Coupling biochemical profiling with molecular fingerprinting provides a practical tool for nutritional valorization, cultivar authentication, and prevention of food fraud or substitution.

## 1. Introduction

Common bean (*Phaseolus vulgaris* L.) is the most widely consumed legume worldwide, with a global production exceeding 30 million tons in 2024. The largest dry-bean producers are Asia (over 14 million tons), followed by Africa (~8 million tons) and South America (~3 million tons), a ranking that has remained unchanged over 2019–2024 according to FAOSTAT data [1].

Common bean is a versatile crop cultivated for both its dry seeds and fresh pods and is available on the market in various forms, including fresh, frozen, canned, and dehydrated products. Green beans are a widely traded fresh vegetable, with the European Union representing a substantial and stable import market [2]. The green pod has a high-water content, elevated dietary fiber, and lower protein and starch on a fresh-weight basis, and its nutritional quality is strongly modulated by genotype, morphotype, and maturity stage at harvest [3,4,5]. The present study concerns the green pod, positioning it as a nutrient-rich vegetable and a raw material for food applications.

The nutritional relevance of common bean lies in its richness in protein, carbohydrates, fibers, starch, vitamins (A, B6, C, E, K), and minerals [6,7,8,9,10,11]. It is also one of the most sustainable protein sources available [6], which underpins its growing use as a raw material for functional foods and plant-based ingredients.

In dried beans, globulins and albumins constitute the major protein fractions (50–70% of total protein), with phaseolin—the predominant globulin, a mannose-rich glycoprotein—accounting for approximately 40–50% of total seed protein [9]; reported crude protein ranges from 16 to 33% [9] and 16.62 to 23.10% [10]. Green pods are comparably protein-rich on a dry-matter basis: 18.2–31.7% DW by the Kjeldahl method in Croatian landraces [3] and 16.1–23.1% DW in freeze-dried French bean pods [4], whereas soluble protein determined by the Bradford method in Mexican landraces was lower (7.0–12.2% DW) [5], showing that the difference reflects the analytical approach (total nitrogen vs. soluble protein) rather than a true compositional gap.

Carbohydrates—mainly starch, non-starch polysaccharides, and derivatives such as oligosaccharides—are the predominant fraction in dried beans, at 55–65% DW [9]; in dry seeds of Himalayan landraces, total carbohydrate ranged from 22.67–37.76% [10]. In green pods, total carbohydrate is likewise the major fraction, reported at 62.0–70.6% DW in freeze-dried French bean pods [4], while whole borlotto pods contain about 20% carbohydrate on a fresh-weight basis, roughly half of which is dietary fiber [12]. Starch, dietary fiber, and non-starch polysaccharides act as fermentable substrates for the gut microbiota, supporting intestinal health [9].

Dried beans are a rich source of dietary fiber, including cellulose, hemicellulose, pectin, oligosaccharides, and lignin. Crude fiber in dry seeds has been reported at 5.71% [7]. Green pods are notably fiber-rich: 7.7–11.1% DW in Croatian landraces [3], with total fiber reaching 9.3 g/100 g FW in borlotto pods—approximately half of their carbohydrate content [12]—consistent with the structural cell-wall material of the pod wall, which increases as the pod matures. Dietary fibers regulate carbohydrate release during digestion [10]; crude fiber correlates significantly with cellulose within the fiber fraction, and transcriptome–metabolome analyses have linked starch/sucrose metabolism and secondary-metabolite biosynthesis (UDP-glucose, ethylene, jasmonic acid, and flavonoids) to cellulose synthesis [13].

Green pods are especially low in fat—0.46 g/100 g FW in borlotto pods [12] and 0.1–1.1% DW in French bean pods [4]—yet the pod lipid fraction is nutritionally favorable, being dominated by monounsaturated fatty acids (~52% of total FAME, chiefly oleic acid), followed by saturated and polyunsaturated fatty acids [12]. Fatty acids support cardiovascular function, immune response, tissue development, and the prevention of obesity and dyslipidemia [9].

The predominant macro elements in common beans are calcium (Ca), magnesium (Mg), and potassium (K), with Ca showing greater bioavailability than K or Mg [9]. These elements are relevant in the prevention of Alzheimer’s disease, type 2 diabetes, and cardiovascular disease [14,15]. Beans also supply essential trace elements—copper (Cu), manganese (Mn), selenium (Se), zinc (Zn), and iron (Fe)—involved in immune function and potential cancer-risk reduction [9]. Green pods contribute meaningfully to mineral intake: K, Ca, Mg, and P have been quantified in snap bean pods (e.g., K 21.7, Ca 9.64, P 5.78, Mg 4.5 g kg^−1^ DW) [16] and Ca, K, and P in Croatian pod landraces [3]. This dietary contribution can be expressed against nutrient reference values (NRVs) [17], providing a food-labeling framework for the mineral density of pod-based products.

Beyond macronutrients, beans contain phenolic compounds and other phytochemicals [18]. The most abundant are phenolic acids, flavonoids, coumarins, and proanthocyanidins [19,20]. Flavonoids exhibit antioxidant, antiviral, antimicrobial, antidiabetic, and anti-inflammatory properties and inhibit lipid peroxidation [21,22]. In dry seeds, total phenolic content ranges from 4.24 mg GAE g^−1^ DW [7] to 14.05 mg GAE g^−1^ DW [10], and flavonoids from 1.99 mg CE g^−1^ DW [7] to 9.26 mg QE g^−1^ DW [10]. Green pods are themselves a relevant source of phenolics: total polyphenols in *Phaseolus vulgaris* pods ranged from 4.2–10.1 mg GAE g^−1^ DW, with pigmented (reddish/purple) landraces showing the highest concentrations and antioxidant activity [5].

Phenolic compounds act as antioxidants by inhibiting radical formation, suppressing ROS-generating enzymes, chelating iron and copper, or acting as hydrogen-donating radical scavengers [18,23]. They also exert antimutagenic, anti-inflammatory, and anticarcinogenic effects [18,19] and, in planta, contribute to cell-wall structure, growth regulation, and stress defense [21]. Although phenolic acids and tannins are commonly classified as antinutritional compounds, they also possess antioxidant, anticarcinogenic, and other bioactive properties that may contribute to the health benefits associated with bean consumption [9,24,25]. Dietary polyphenols are largely unabsorbed in the small intestine and are metabolized by colonic microflora; the food matrix strongly modulates their bioavailability, since proteins and carbohydrates interact with polyphenols and reduce absorption [26].

Within a circular-economy framework, bean by-products—seed coats, *Phaseoli peri-carpium*, damaged seeds, and processing wastewater—are valorized in functional foods, nutraceuticals, and natural-colorant formulations, exhibiting antioxidant, antidiabetic, diuretic, anti-inflammatory, and antibacterial activities [27]. The pod itself is the main biomass by-product of bean processing: borlotto bean pods, though usually discarded, are a low-calorie matrix rich in dietary fiber, protein, and polyphenols (approximately 570 mg GAE per 100 g dry extract, ~20% of which are flavonoids), with significant antioxidant and anti-inflammatory activity, supporting their valorization as a sustainable source of food-grade functional ingredients [12].

Reliable identification and authentication of food-grade germplasm require molecular tools alongside compositional data. The common bean is a self-pollinated diploid (2n = 2x = 22) with a 473 Mb genome, domesticated independently from Andean and Mesoamerican wild gene pools, and today cultivated across the Americas, Europe, Africa, and Asia; substantial genetic variation, including in mineral content, has been reported [14]. Inter-Simple Sequence Repeat (ISSR) markers are efficient, highly polymorphic tools for assessing genetic diversity and for cultivar fingerprinting, supporting traceability and the prevention of food fraud or substitution.

However, despite the high nutritional and economic importance of *Phaseolus vulgaris* L., common bean production faces severe challenges due to current climate variability, which directly threatens pod development and nutritional consistency. While breeding programs continuously generate novel lines with outstanding phenotypic characteristics—such as extreme pod dimensions or distinct pigmentation—there is a critical knowledge gap in the existing literature regarding their inter-annual stability across fluctuating growing seasons. Most studies evaluate biochemical profiles under isolated, single-year conditions, leaving a fundamental question unanswered: do these newly developed lines maintain their superior nutritional and bioactive properties over time, or are their traits highly unstable under environmental shifts?

To address this limitation, we hypothesized that combining multi-seasonal biochemical screening with high-resolution molecular fingerprinting can accurately identify stable, elite germplasm tailored for sustainable agriculture and successful food acceptance. Therefore, the primary objective of this study was to evaluate the physical, nutritional, and bioactive profiles of three newly developed *Phaseolus vulgaris* L. genotypes (L HELD, L 301B, and L BEC) over two consecutive cultivation years (2024–2025), focusing on key attributes that may influence consumer palatability and culinary processing behavior. Concurrently, Inter-Simple Sequence Repeat (ISSR) molecular markers were employed to clarify the underlying genetic diversity and polymorphism among these lines. By aligning dynamic chemical transformations and dietary traits with stable molecular patterns, this research provides a comprehensive blueprint for selecting climate-resilient cultivars capable of supporting global food security, functional nutrition, and consumer-approved food options.

## 2. Materials and Methods

### 2.1. Reagents and Chemicals

The following analytical-grade reagents were used: acetone ≥ 99.8% (Merck KGaA, Darmstadt, Germany); gallic acid ≥ 98% (Carl Roth GmbH + Co. KG, Karlsruhe, Germany); Folin–Ciocalteu phenol reagent 2 N (Sigma-Aldrich Chemie GmbH, Taufkirchen, Germany); sodium carbonate 99.8% (Merck KGaA, Darmstadt, Germany); rutin trihydrate ≥ 95% (Carl Roth GmbH + Co. KG, Karlsruhe, Germany); sodium nitrite 99% (Carl Roth GmbH + Co. KG, Karlsruhe, Germany); aluminum chloride hexahydrate ≥ 99% (Honeywell International, Inc. Fluka^TM^, Morris Plains, NJ, USA); 2,2-diphenyl-1-picrylhydrazyl (DPPH, 95%, Alfa Aesar, Thermo Fisher Scientific, Kandel, Germany); Trolox 97% (Acros Organics, Thermo Fisher Scientific, Geel, Belgium); hydrogen peroxide 30%, Suprapur^®^ (Merck KGaA, Darmstadt, Germany); and nitric acid 69%, ROTIPURAN^®^ Supra (Carl Roth GmbH + Co. KG, Karlsruhe, Germany), InnuPREP Plant DNA I Kit-IPC16 (Analytik Jena GmbH, Jena, Germany), SYBR™ Safe DNA Gel Stain (Invitrogen, Carlsbad, CA, USA).

### 2.2. Plant Material

**Figure 1:**
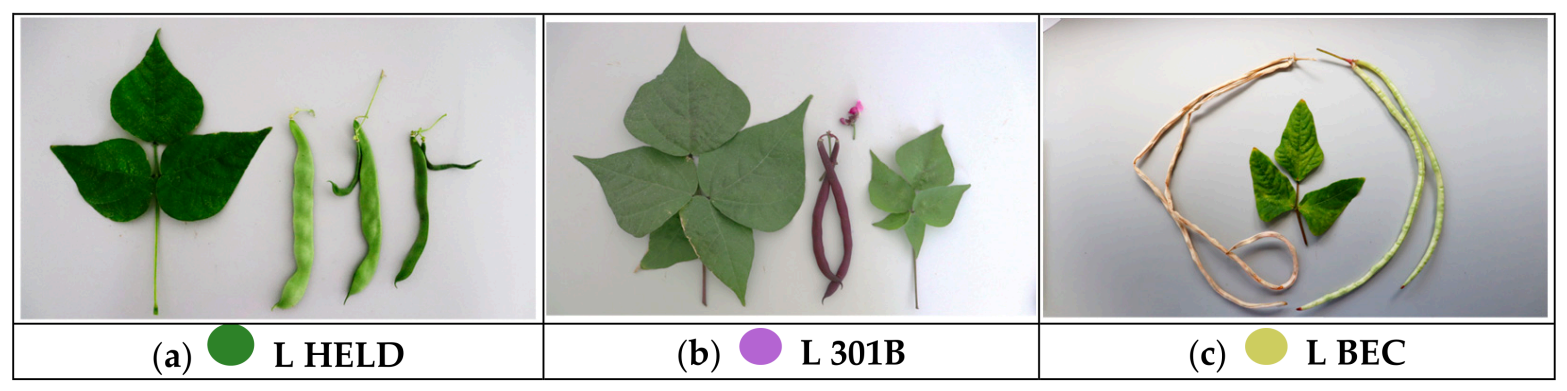
Fresh bean pod genotypes. (**a**) Fresh pods and leaf of L HELD genotype, (**b**) fresh pods and leaf of L 301B genotype, (**c**) fresh and dry pods and leaf of L BEC genotype. The color of the bullets, respectively dark green for L HELD, purple for L 301B, and yellow-green for L BEC, will be the code color for each genotype in this study.

The analyzed genotypes (L HELD, L BEC, and L 301B) are part of the germplasm collection of the Plant Genetic Resources Bank for Vegetable, Floriculture, Aromatic and Medicinal Plants Buzău (BRGV Buzău), which includes approximately 60 lines of the *Phaseolus vulgaris* species. They were cultivated in polytunnel greenhouses at BRGV Buzău (45°08′50.7″ N, 26°47′55.7″ E). The genotypes are characterized by distinctness, uniformity, and genetic stability, representing valuable genetic resources for the conservation and utilization of the genetic diversity of *Phaseolus vulgaris* L. From a culinary and processing perspective, L HELD was selected for its broad, flat, stringless pods with absent or very weak curvature, resulting in straight pods with high commercial appeal and suitability for processing and packaging. L 301B was selected for its dark violet pods, which provide an attractive and distinctive color profile for the fresh food market. Finally, L BEC was selected for its exceptional pod length, a distinctive agronomic trait that provides high commercial potential through increased visual attractiveness and high yield per pod. Its pods exhibit a bicolored pattern, with a green primary background and purple secondary pigmentation. This distinctive coloration undergoes an ontogenetic change during maturation, from green to purple-black and finally beige at the dry stage, providing harvesting flexibility at different developmental stages for either fresh snap bean or dry grain production. Furthermore, its black, reniform (kidney-shaped) seeds represent a commercially valuable trait particularly suited to the dry bean market (Figure 1).

Pods were harvested at consumption maturity over two growing seasons, on 19 September 2024 and 19 August 2025. Fresh pods were transported the same day at 4 °C to the Research Center for the Study of Food and Agricultural Products Quality (University of Agronomic Sciences and Veterinary Medicine of Bucharest) for physicochemical analysis (Figure 1), and stored at −80 °C until analysis. Young fresh leaves were collected separately for ISSR molecular analysis and handled identically. Except for total soluble solids (°Brix), which was measured on the fresh product, all biochemical assays, titratable acidity, bioactive compounds, FT-NIR proximate composition, and mineral contents were expressed on a dry-weight basis (DW) to remove moisture-variation artifacts and ensure comparability across genotypes and years.

The content of dry matter, total soluble sugars, titratable acidity, total polyphenols, total flavonoids, antioxidant capacity, assimilatory pigments, proteins, fiber, ash, sugars, and starch in pods was determined as follows.

### 2.3. Morphometric Traits

Pod weight and pod length were determined using a Radwag PS 6000.R2 high-precision electronic laboratory balance (RADWAG Wagi Elektroniczne, Radom, Poland), and a standard graduated ruler (accuracy: ±1 mm), respectively. The results were expressed as g and cm, respectively (mean ± SD).

### 2.4. Dry Matter, Total Soluble Solids, and Titratable Acidity

Dry matter (DM) was determined gravimetrically by drying 1 g of homogenized fresh pod samples in weighing bottles (Ø 50 × 30 mm) at 105 °C to constant weight (Memmert oven, Memmert GmbH + Co. KG, Schwabach-Uigenau, Germany) [28].

Total soluble solids (TSS) were determined using a Krüss DR301-95 digital handheld refractometer (Krüss Optronic GmbH, Hamburg, Germany), from the freshly squeezed juice of the fresh pods. Results were expressed in °Brix [29,30].

Titratable acidity (TA) was determined using a TitroLine^®^ automatic titrator (SI Analytics, Mainz, Germany). Samples were titrated with 0.1 N sodium hydroxide to pH 8.1. Results were expressed as g citric acid/100 g DW [31,32].

### 2.5. Asimilatory Pigments Assay

Chlorophyll a (Chl a), chlorophyll b (Chl b), and total carotenoids were extracted from fresh samples with 80% acetone (1:50, *w*/*v*), filtered under reduced pressure, and quantified on a Specord 210 Plus UV-VIS spectrophotometer (Analytik Jena GmbH + Co. KG, Jena, Germany) at 663, 646, and 470 nm [33]. The results were expressed as mg/100 g DW.

### 2.6. Total Phenolic Content, Total Flavonoid Content, and Antioxidant Capacity Assay

Extraction. One gram of fresh sample was mixed with 10 mL of 70% methanol and left overnight in the dark at room temperature. Samples were then shaken for 60 min. (orbital shaker KS260C, IKA-Werke GmbH & Co. KG, Staufen, Germany), centrifuged (5000 rpm, 5 min, 4 °C; 5430R, Eppendorf SE, Hamburg, Germany), and the supernatant was collected. The pellet was re-extracted twice with 10 mL of 70% methanol; the three supernatants were combined.

Total polyphenol content (TPC). TPC was determined by the Folin–Ciocalteu method: 0.5 mL extract or gallic acid standard + 2.5 mL Folin–Ciocalteu reagent 2 N (1:10); after 2 min, 2 mL of 7.5% Na_2_CO_3_ was added; samples were vortexed, incubated in a water bath at 50 °C for 15 min, cooled on ice, and read at 760 nm (calibration 0–100 µg/mL). Results were expressed as mg GAE/100 g DW. The results were expressed as milligrams of gallic acid equivalents per 100 g DW (mg GAE/100 g DW) [34].

Total flavonoid content (TFC). TFC was determined using the aluminum chloride method. Reaction mixture: 750 µL extract or rutin standard + 750 µL distilled water + 90 µL 5% NaNO_2_. After 5 min (in the dark, at room temperature), 90 µL of 10% AlCl_3_ was added. After another 5 min, 600 µL of 1 M NaOH and 720 µL of distilled water were added. Absorbance was read at 510 nm. Calibration curve: 0–30 µg/mL. The results were expressed as milligrams of rutin equivalents per 100 g DW (mg RE/100 g DW) [35].

Antioxidant Capacity (DPPH). Antioxidant capacity was determined using the DPPH method. Reaction mixture: 2 mL 0.2 mM DPPH solution + 250 µL extract or Trolox standard. Incubation: 30 min in the dark. Absorbance: 515 nm. Calibration curve: 0–4 mM. The results were expressed as milligrams of Trolox equivalents per 100 g DW (mg TE/100 g DW) [36].

### 2.7. Macro- and Micronutrient Profile by ICP-MS

Macro- and micronutrient extraction was performed using 0.250 g fresh samples, followed by microwave digestion with 2 mL H_2_O_2_ solution 30%, Suprapur^®^ (Merck KGaA, Darmstadt, Germany), and 8 mL HNO_3_ ROTIPURAN^®^Supra 69% (Carl Roth GmbH + Co. KG.), using the ETHOS UP microwave digestion system (Milestone S.r.l., Sorisole (BG), Italy). For microwave digestion, the following parameters were used: time 1 with 15 min, 1800 MW(W), T1 180 °C, T2 100 °C; time 2 with 20 min, 1800 MW(W), T1 180 °C, T2 100 °C, and at the end of the program, cooling time 15 min. After extraction, the samples were diluted to 50 mL with ultrapure water (resistivity of 18.2 MΩ) obtained by Milli-Q equipment (Merck KGaA, Darmstadt, Germany). Analysis was performed using ICP-MS on a 7700x Agilent Technologies (Agilent Technologies, Inc., Santa Clara, CA, USA). Helium (He) was used as the collision gas in He mode. Calibration used multielement standards (1000 µg/mL each of Ca, Fe, K, Mg, Na; 10 µg/mL each of Ag, Al, As, Cd, Co, Cr, Cu, Mn, Mo, Ni, Pb, Se, Zn in 5% HNO_3_); calibration ranges were 1–8 ppm for microelements and 100–800 ppm for macroelements [35,37]. Macroelement concentrations were expressed as mg/100 g DW and microelement concentrations as mg/kg DW.

The contribution of each genotype to dietary mineral intake was evaluated as the percentage of the nutrient reference value (%NRV) established in [17], Annex XIII, using the following reference values: K 2000 mg, Ca 800 mg, Mg 375 mg, Cu 1 mg, Mn 2 mg, Zn 10 mg. The %NRV was calculated per 100 g of fresh pod (edible portion, as consumed), according to the equation:

(1) $$\%\mathrm{NRV}=\left(\frac{\mathrm{mineral}\;\mathrm{content}\;\mathrm{per}\;100\;g\;\mathrm{FW}}{\mathrm{NRV}}\right)\times 100$$

The raw data for micronutrients (Mn, Cu, and Zn), originally expressed in mg/kg, were converted to mg/100 g by dividing the values by 10. Regarding sodium, the values were converted to their salt equivalent (NaCl) by multiplying the sodium content by 2.5, as Regulation (EU) No. 1169/2011 [17] establishes a reference intake for salt (6 g) rather than sodium alone.

### 2.8. Proximate Composition by FT-NIR

Protein, fiber, ash, sugars, and starch were determined by FT-NIR spectroscopy. Samples were lyophilized and ground to a particle size of 0.5 mm, transferred to transparent glass vials (52 × 22/1.20 mm), and scanned from 3600 to 12,500 cm^−1^ using the FT-NIR Multi-Purpose Analyzer (Bruker Optics, Ettlingen, Germany) with OPUS 7.5 software and dedicated calibration software, Ingot Specman Gold (Ingot L4 Feed & Ingredients—legumes). Scanning was performed in absorbance mode using the integrating sphere, and each spectrum represented an average of 64 scans. Background spectra were also collected using 64 scans. The wavelength resolution was 8 cm^−1^. The method was adapted from Wafula et al. (2028) [38]. The results were expressed as % DW.

### 2.9. Molecular Markers Analysis

#### 2.9.1. DNA Extraction

Extraction of genomic DNA was performed using 70–90 mg of young leaves for L 301B, I HELD, and L BEC bean genotypes. Genomic DNA was extracted using an automated extraction system (InnuPure C16, Analytik Jena GmbH, Jena, Germany) based on the principle of magnetic particle separation for fully automated DNA isolation and purification. InnuPREP Plant DNA I Kit-IPC16 (Analytik Jena GmbH, Jena, Germany) was used for genomic DNA extraction following the manufacturer’s instructions. A preliminary manual processing step was the external lysis of the starting material. The plant sample was ground in the presence of liquid nitrogen to a fine powder and homogenized with SLS lysis solution (containing CTAB as a detergent component), Proteinase K, and RNase A solution. After external lysis, the extraction proceeded with automated DNA extraction using the Ext Lysis_200_C16_04 program. The DNA was quantified at 260 nm, and its purity was measured at a 260 nm/280 nm absorbance ratio. All measurements were conducted with a NanoDrop TM1000 spectrophotometer (Thermo Fisher Scientific, Waltham, MA, USA). DNA integrity was verified via horizontal 1% agarose gel electrophoresis in 1× TAE buffer stained with SYBR Safe (Invitrogen). Samples mixed with 10× loading buffer were visualized using a PharosFX Molecular Imager (Bio-Rad, Hercules, CA, USA), confirming intact genomic DNA fragments.

#### 2.9.2. ISSR Analysis

Five anchored ISSR primers consisting of di- and tri-repeat motifs were selected (Table 1) and synthesized by ANTISEL/CeMIA SA (cellular and molecular immunological applications, GR.) for screening in this study. These anchored primers have an extended portion of bases at the 5′ or 3′ end of their sequence to increase the specificity of the amplicon, such as polymorphic content, and their capability of distinguishing between genotypes. Total volume for the PCR reactions was 25 µL, containing Platinum™ II Hot-Start PCR Master Mix (2× with Platinum™ II Taq Hot-Start DNA Polymerase premixed in an optimized PCR buffer with dNTPs and 1.5 mM MgCl2 in final reaction concentration (Invitrogen™, Carlsbad, CA, USA), 0.8 µmol/µL primer, 5 µL Platinum™ GC Enhancer, and 50 ng genomic DNA. Amplification was performed with a Mastercycler^®^ Nexus system (Eppendorf™SE, Hamburg, Germany) as follows: one cycle at 94 °C for 2 min, 35 cycles at 94 °C for 15 s, 51 °C for 30 s, 68 °C for 1 min, and a final extension of 68 °C for 2 min. Amplification products were separated in 1.5% agarose gels (TopVision-Thermo Scientific™, Waltham, MA, USA) using 200 bp Ladder (Carl Roth^®^, Karlsruhe, Germany) and 1 kb Plus DNA Ladder (Invitrogen™, Carlsbad, CA, USA) as references. Gels were stained with 1× SYBR™ Safe DNA Gel Stain (Invitrogen™, Carlsbad, CA, USA) in 1× TAE buffer following a conventional protocol for electrophoresis. Agarose gels were scanned with a molecular imager PharosFX™ Plus system at 488 nm and provided with an external laser for high-resolution and precise spectral assignment.

### 2.10. Statistical Analysis

Statistical analyses and graphics were performed using R version 4.3.3 with the pheatmap, reshape2, ggplot, and ggrepel packages. Data were subjected to a one-way ANOVA followed by Tukey’s HSD test (*p* < 0.05). Statistical comparisons were carried out independently for each individual parameter (column-wise). Different letters (a, b, c, d) indicate statistically significant differences among variety–year combinations.

Relationships among physicochemical, nutritional, and bioactive parameters were examined by Pearson correlation analysis; correlation coefficients (r) were calculated pairwise and visualized as a correlation matrix (heatmap). Multivariate structure was explored by Principal Component Analysis (PCA) on standardized/scaled physicochemical and nutritional variables, with genotype–year groups displayed as confidence ellipses in the PC1–PC2 space. Pairwise genetic similarity analysis was performed in RStudio program version 4.4.1.

## 3. Results

### 3.1. Morphological and Quality Parameters Characterization of Bean Genotypes

#### 3.1.1. Pod Weight and Length

Pod weight and length are crucial commercial traits that directly influence both crop yield and consumer preferences. In the present study, pod weight varied among genotypes from 5.03 to 32.53 g, while pod length ranged from 11.06 to 82.88 cm. L BEC exhibited the highest pod weight and pod length across both study years, with mean values of 30.16 g and 70.94 cm, respectively (Table 2). Similar ranges for pod traits in common bean genotypes have been reported in the literature; for instance, ref. [39] observed pod lengths between 12.69 and 17.35 cm and pod weights between 5.51 and 8.63 g. Likewise, Bhattacharjee et al. (2023) [40] reported pod lengths of 3.42–15.55 cm and pod weights of 4.64–6.70 g, and García Fernández et al. (2023) [41] found variation between 7.5 cm and 26.5 cm for pod length, values that are directly comparable to those recorded for L HELD and L 301B in our study. L 301B was found to be the most stable genotype, exhibiting no significant differences between the two study years (Table 2).

#### 3.1.2. Dry Matter

Dry matter (DM) content is a critical indicator of pod density, texture, and post-harvest stability [42]. In the present study, DM ranged from 9.20% to 17.95%, with the highest annual values recorded in lines L HELD and L 301B during the 2025 cultivation year. Line L 301B also exhibited a robust overall accumulation, reaching a mean DM value of 15.21% (Table 2). In common bean, increased dry matter partitioning to pods and seeds, together with efficient water use, has been associated with improved terminal drought resistance [43].

#### 3.1.3. Titratable Acidity (TA)

TA is a key parameter influencing product stability and flavor balance. Beyond flavor balance, organic acids may also support mineral bioavailability and central metabolic processes, while their reported health-related effects include anti-inflammatory activity, improved calcium absorption, and modulation of intestinal and immune functions [44]. In our study, TA varied between 0.76 g/100 g and 1.92 g/100 g, with the highest annual value recorded in L BEC 2025. L BEC also showed the highest overall mean TA, 1.71 g/100 g (Table 2). No statistically significant differences were found between L HELD and L 301B across cultivation years. Similar results have been found by Aquino-Bolaños et al. (2021) [5] of 0.6–1.2 TA.

#### 3.1.4. FT-NIR

Ash content

Ash content reflects the total mineral fraction, contributing significantly to the nutritional value of the pods. In the present study, ash values varied between 4.91% and 6.84%, with L HELD 2025 exhibiting the highest mineral residue. Line L 301B recorded the highest overall mean ash content at 6.50%, while also accumulating the highest dry matter (DM) content (15.21%) (Table 2). As L 301B proved to be the most stable genotype, no significant differences were observed between the study years, a trend consistent with the findings for the pod weight parameter. Our results are in agreement with those reported by Sinkovič et al. (2024) [4], who found ash contents ranging from 4.52% to 8.21%. Beyond its nutritional relevance, ash content provides an estimate of the total inorganic fraction of plant tissues and may reflect differences in mineral accumulation among genotypes. In common bean and other edible-podded legumes, variation in ash content has been associated with differences in the concentrations of nutritionally important minerals, including potassium, calcium, magnesium, phosphorus, and trace elements [45].

Total fiber content

Total fiber content is an important functional component associated with digestive health and nutritional quality [8]. Dietary fibers can help manage diabetes by reducing blood glucose levels; indeed, it has been reported that patients with non-insulin-dependent diabetes could reduce their need for insulin by up to 40% by incorporating green beans into their regular diet [5]. In the present study, total fiber content ranged from 7.99% to 15.47%, with the highest annual values observed in line HELD in 2025 and line 301 B in 2025 (Table 2). The highest overall mean fiber content was recorded in line 301 B, at 14.55%. Line 301 B proved to be the most stable genotype, showing no significant differences between the study years, a trend consistent with the findings for pod weight and ash parameters.

Total protein content

Given the growing interest in common beans as primary raw materials for novel plant-based protein ingredients [8,11], the protein contents were evaluated. In the present study, protein content ranged from 26.33 ± 0.04% to 37.50%, with the highest annual value recorded in L BEC in 2025. L BEC also exhibited the highest overall mean protein content, at 34.74% (Table 2). Line 301 B was the most stable genotype, displaying no significant inter-annual variation, a pattern similar to that observed for pod weight, ash, and fiber accumulation. The values are higher than those reported by Bhattacharjee et al. (2023) [40] (18.90–20.10%) and Menga et al. (2023) [46] (16.82–26.60%). However, our results align more closely with the ranges reported by Vieler et al. (2026) [11] (20–30%) and Carović-Stanko et al. [3] (18.2–31.7%). As noted by Bernardi et al. (2026) [9], common bean protein content typically falls between 16% and 33%, being modulated by genotype, environmental and cropping conditions, cultivation year, geographical location, climate, soil characteristics, and fertilization practices. Consequently, enhancing bean protein accumulation supports the dietary shift toward sustainable, plant-based nutrition, providing highly digestible amino acids necessary for maintaining human metabolic homeostasis without the environmental footprint of animal proteins.

Starch

Nutritionally, bean starch is rich in resistant starch that escapes small intestine digestion and undergoes bacterial fermentation in the colon, acting as a prebiotic that fuels beneficial gut microbiota and generates health-promoting short-chain fatty acids. Also, starch contributes to the energy value, texture, and culinary behavior of the pods. Starch characterization is crucial due to the high content of nitrogen-free extract in the coproducts, according to Espinosa-Alonso et al. (2025) [8]. In the present study, starch content ranged from 24.81% to 43.51%, with the highest annual value recorded in L HELD 2024. The highest overall mean starch content was also found in L HELD, 36.16% (Table 2). As L 301 B and L BEC were the most stable genotypes, no significant differences were found between the study years.

Total sugar content

Total sugar content influences sweetness perception and may contribute to the sensory quality and consumer acceptability of green bean pods [47]. In the present study, total sugar content ranged from 6.76% to 17.40%, with the highest annual value recorded in L BEC 2025. L BEC also exhibited the highest overall mean sugar content, 15.83%. In contrast, L HELD showed no significant differences between cultivation years, indicating a stable sugar profile (Table 2). Our results are higher than those reported by Bhattacharjee et al. (2023) [40] (4.7–6.35%), but were within the ranges reported by Carović-Stanko et al. (2026) [3], who found total sugar content in green bean pods varied from 11.3% to 28.6%. The contribution of sugars to green bean quality is supported by previous studies showing that variation in fructose, glucose, and sucrose among snap bean genotypes can influence pod flavor and consumer acceptance [47].

#### 3.1.5. Assimilatory Pigments

Chlorophyll is an essential photosynthetic pigment located in the chloroplasts of higher plants [48] and is abundantly found in green fruits and vegetables that form an integral part of the human diet [48,49]. Food color—both naturally and during processing—directly influences consumer acceptability and appetite, while also serving as a simple indicator for evaluating food quality [50]. Besides their role in coloration, these pigments exhibit antioxidant, antitumoral, antiviral, and anti-inflammatory properties, thereby offering significant health benefits [51]. Photosynthetically active pods protect and nourish developing seeds, exhibiting photosynthetic rates up to 3–15% in legumes [52]. During maturation, pod tissues increase carbohydrate and amino acid synthesis, with pericarp chloroplasts differentiating into amyloplasts to store excess sugars as starch [53,54]. The chlorophyll a/b ratio in green snap bean (*Phaseolus vulgaris*) fruits serves as a primary biochemical indicator of pod photosynthetic competence, developmental maturity, and post-harvest or stress-induced senescence. Ultimately, chlorophyll degradation drives pod degreening and color changes, though the molecular mechanisms in the common bean remain poorly understood [48].

**Figure 2:**
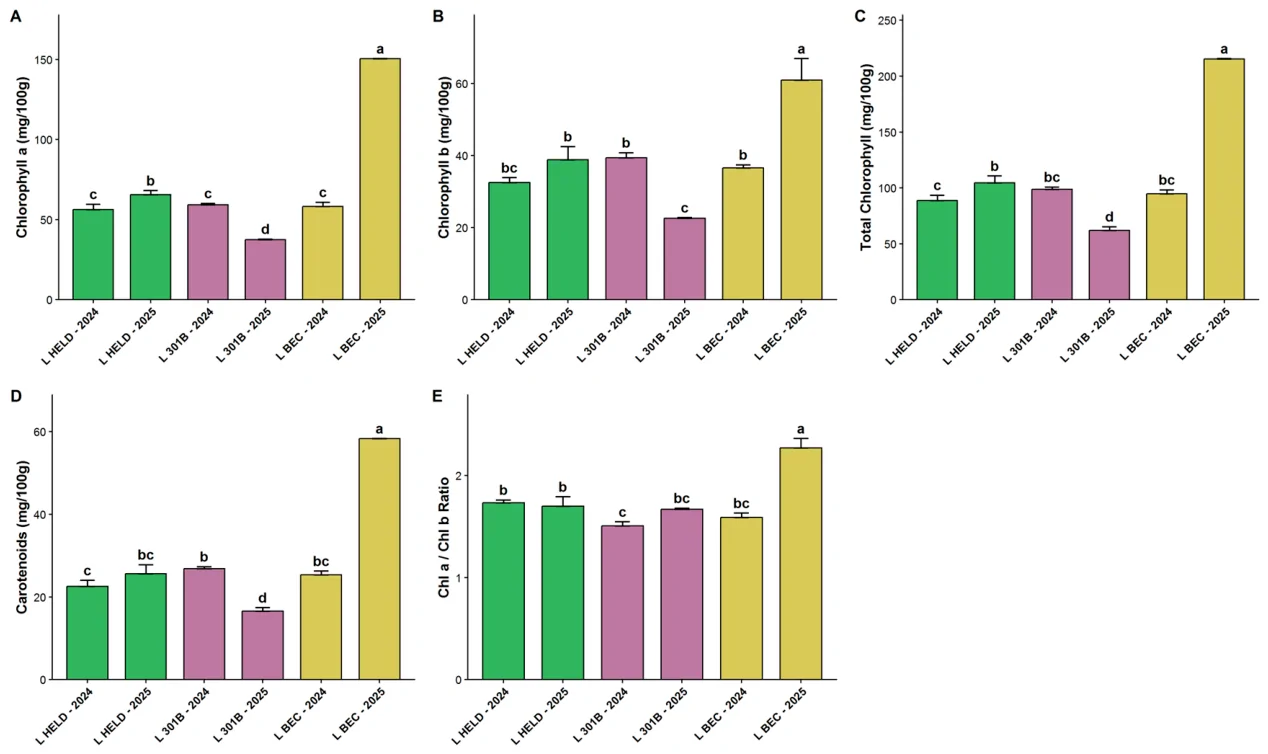
Assimilatory pigments in bean genotypes. (**A**) Chloropyll a; (**B**) Chloropyll b; (**C**) Total Chloropyll; (**D**) Total carotenoid content; (**E**) Chl a/Chl b ratio. Data are presented as Mean ± Standard Deviation (*n* = 3). Different letters above each bar within the same graph indicate statistically significant differences according to Tukey’s HSD test (*p* < 0.05). Comparisons were performed independently for each parameter.

In our study, Chl a content varied between 36.91 mg/100 g for L301 B in 2025 and 150.59 mg/100 g for L BEC in 2025 (Figure 2A). Chlorophyll b varied from 22.43 mg/100 g for L 301 B in 2025 to 65.17 mg/100 g for L BEC in 2025. The highest value was recorded in 2025 for the L BEC genotype, which was correlated with the lowest dry matter content (Figure 2B). Regarding the Chl a/Chl b ratio, statistically significant differences were observed between the L HELD and L BEC genotypes overall. Individually, the L HELD genotype demonstrated high interannual stability with no significant variation between 2024 and 2025, whereas the L BEC genotype exhibited significant differences between the two study years (Figure 2E).

The analysis of total carotenoid and total chlorophyll content revealed a significant genotype-by-year interaction, highlighting how annual growing conditions influence secondary compound accumulation in the studied *Phaseolus vulgaris* L. lines (Figure 2C,D). In 2025, genotype L BEC exhibited an outstanding accumulation of carotenoids (~58 mg/100 g), which was statistically superior to all other lines. Conversely, L 301B showed a divergent trend, with its carotenoid content dropping significantly from 2024 to 2025, reflecting marked inter-annual variability. In contrast, L HELD demonstrated the highest biochemical stability, maintaining statistically comparable carotenoid levels across both experimental years. These findings suggest that while L BEC possesses the highest genetic potential for carotenoid synthesis, L HELD offers superior resilience across years (Figure 2D).

These results highlight clear functional differences between the two new lines. The significant inter-annual variation in the Chl a/Chl b ratio observed in L BEC demonstrates high inter-annual shifts in the Chl a/Chl b ratio, pointing to dynamic adjustments in pigment ratios across different cultivation years. In contrast, L HELD maintained uniform pigment ratios, demonstrating high multi-year trait stability and predictable performance. Ultimately, the overall significant divergence between L HELD and L BEC at the pigment level strongly correlates with the distinct molecular patterns confirmed by the ISSR analysis, underlining their diverse genetic backgrounds.

#### 3.1.6. Total Phenolic, Total Flavonoid, and Antioxidant Capacity

The rich content and variety of polyphenolic compounds in beans play an important role in antioxidant protection. Consuming beans rich in phenolic and flavonoid compounds—which possess potent antioxidant activity—is associated with a lower incidence of cardiovascular disease, cancer, diabetes, obesity, and neurodegenerative disorders [12,23,42,48,55].

Total polyphenol content varied between 440.46 mg GAE/100 g DW for L HELD in 2025 and 1290.62 mg GAE/100 g DW for L BEC in 2025. Inter-annual stability in TPC accumulation was observed for both L HELD and L BEC, showing no statistically significant differences between the two study years (Table 3).

When compared on a dry weight basis, the TPC values are higher than those reported by Kan et al. (2017) [56] (equivalent to 25–379 mg GAE/100 g DW), but align consistently within the ranges observed by Aquino-Bolaños et al. (2021) [5] (420–2650 mg GAE/100 g) and Nina et al. (2023) [57] (328–1661 mg GAE/100 g DW).

Total flavonoid content (TFC) ranged from 103.18 mg RE/100 g DW for line L BEC in 2024 to 325.71 mg RE/100 g DW for line L 301B, with statistically significant differences detected across both cultivation years and genotypes. Regarding antioxidant capacity, the values followed a similar trend, extending from 5735.73 mg TE/100 g DW for line L HELD in 2024 to 41,590.99 mg TE/100 g DW for line L BEC in 2025. Significant variations were observed for both factors (years and genotypes), indicating high variability similar to the TFC accumulation pattern. Overall, line L BEC exhibited the highest accumulation for both TPC and AC, whereas line L 301B excelled in TFC (Table 3).

The clear separation in bioactive specialization highlights distinct genetic trade-offs among the newly developed lines. The exceptional flavonoid accumulation in line L 301B is strongly coupled with its distinct purple phenotype, reflecting a targeted up-regulation of the anthocyanin biosynthetic pathway [58]. Conversely, the extraordinary antioxidant capacity recorded in line L BEC closely mirrors its maximum total phenolic content, confirming that polyphenols serve as the primary drivers of radical scavenging activity in this genotype. Furthermore, the high inter-annual variability observed for TFC and AC, contrasting with the structural stability of TPC, suggests that while overall polyphenol synthesis is strictly governed by genetic traits, specific flavonoid branching and antioxidant responses are highly dynamic across different cultivation years.

#### 3.1.7. Multi-Elemental Profile

**Figure 3:**
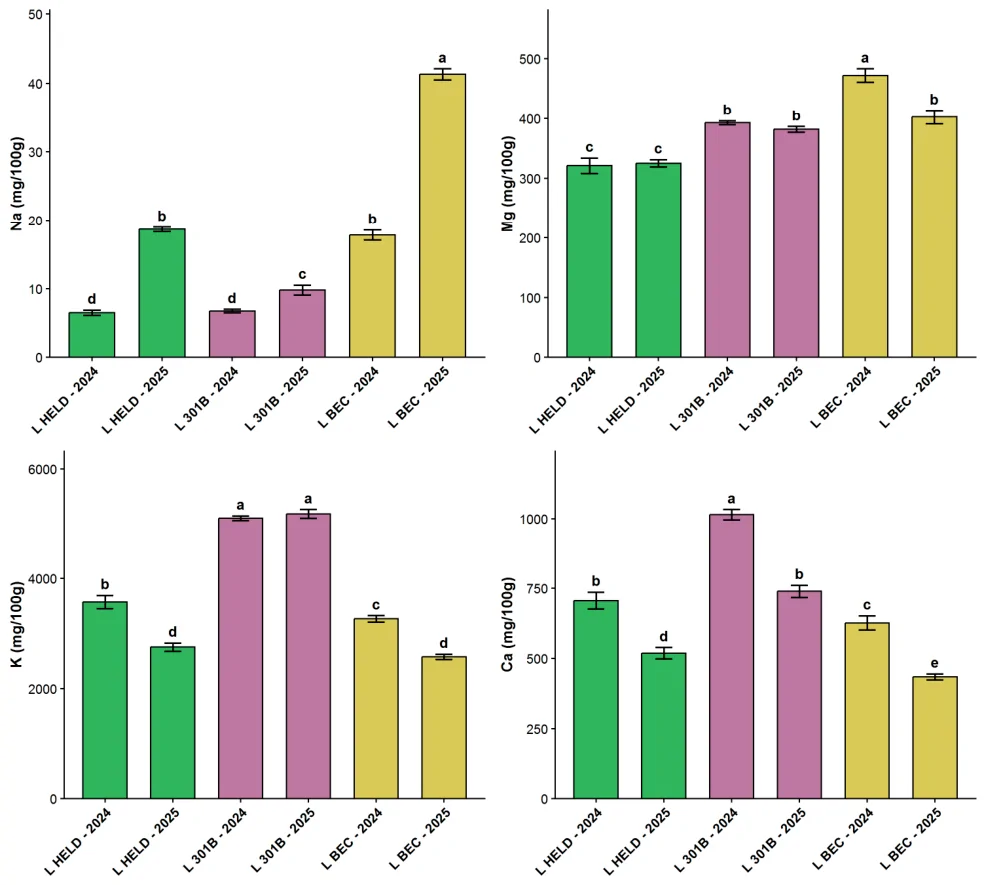
Macroelement content in bean pods. Data are presented as Mean ± Standard Deviation (*n* = 3). Different letters above each bar within the same graph indicate statistically significant differences according to Tukey’s HSD test (*p* < 0.05). Comparisons were performed independently for each parameter.

**Figure 4:**
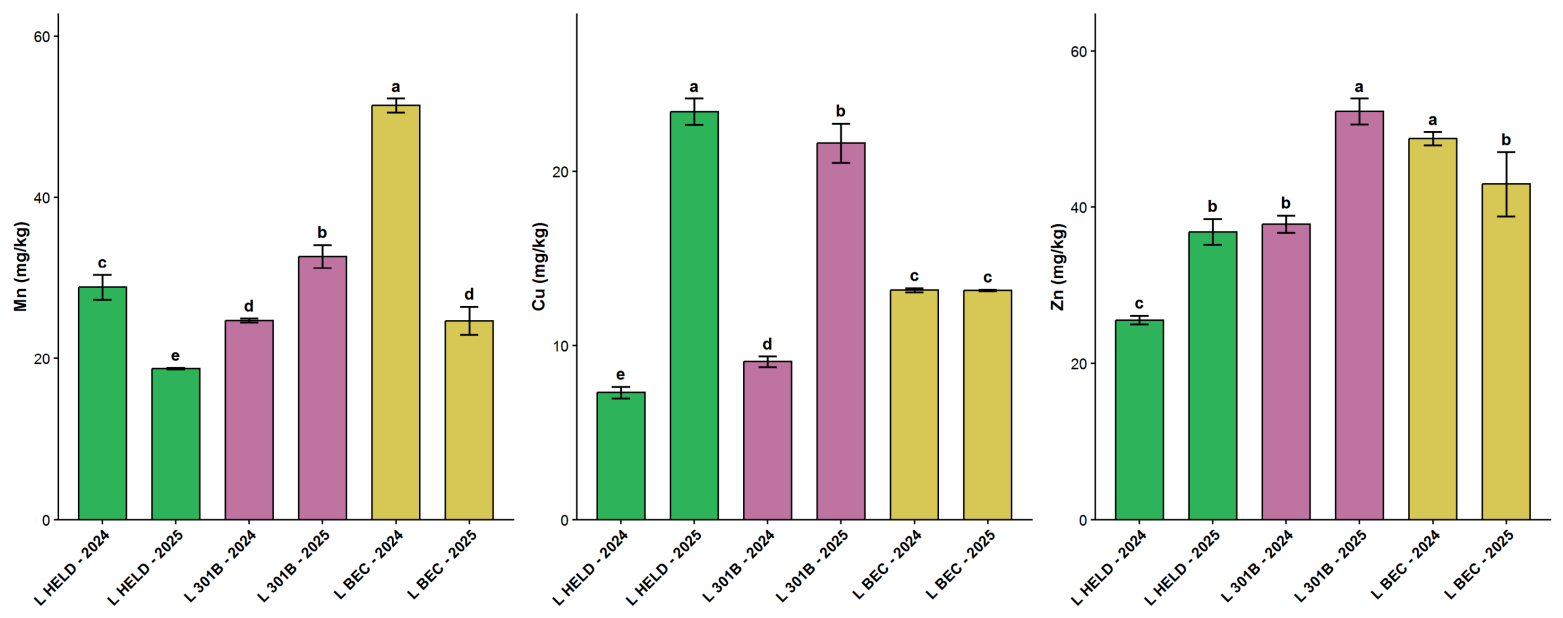
Microelement content in bean pods. Data are presented as Mean ± Standard Deviation (*n* = 3). Different letters above each bar within the same graph indicate statistically significant differences according to Tukey’s HSD test (*p* < 0.05). Comparisons were performed independently for each parameter.

Seven elements found in the pods of bean genotypes were taken into account in this study (Figure 3 and Figure 4). The classification of elements in our study followed Ali et al., who, in their 2023 study, provided multiple classifications in: macro minerals (macro elements), which include sodium (Na), magnesium (Mg), potassium (K) and calcium (Ca), and trace elements (microelements), which include manganese (Mn), copper (Cu) and zinc (Zn).

Macroelements

Macroelements are vital minerals required in substantial amounts to maintain physiological homeostasis. Beyond their structural role in biological tissues, they participate in key metabolic pathways and enzymatic reactions across various physiological systems [59].

In the present study, Na content varied between 6.54 mg/100 g DW for L HELD in 2024 and 41.32 mg/100 g DW for L BEC in 2025, with statistically significant differences observed across both genotypes and cultivation years. Mg content varied from 321.27 mg/100 g DW for L HELD in 2024 to 472.70 mg/100 g DW for L BEC in 2024. Although significant differences were detected among genotypes, no significant inter-annual variation was found, indicating that all analyzed lines displayed high stability in Mg accumulation (Figure 3).

Regarding K, values varied between 2581.18 mg/100 g DW for L BEC in 2025 and 5186.95 mg/100 g DW for L 301B in 2025. Significant genotypic differences were present, with line L 301B being the only genotype demonstrating strict inter-annual stability for K accumulation (Figure 3). These findings regarding K align consistently with those reported by Sinkovič et al. (2024) [4] (originally expressed as 14.81–33.02 g/kg). Furthermore, Ca content varied between 384.79 mg/100 g for L BEC in 2025 and 1014.7 for L 301B in 2024, exhibiting significant fluctuations influenced by both genotype and year, similar to the Na accumulation pattern. Notably, a generalized decrease in both K and Ca content was observed from 2024 to 2025 across all analyzed genotypes (Figure 3).

Microelements

Microelements are essential for plant, animal, and human health, playing crucial roles as vital components of enzymes involved in plant redox reactions and human physiological functions, including the immune and reproductive system regulation [59,60]. In plants, mineral elements are also involved in growth and development, photosynthesis, enzyme activation, nutrient transport, and the regulation of numerous metabolic and physiological processes [61].

Inadequate microelement levels in soil and crops directly impair agricultural productivity and human nutrition, as plant-based foods represent a primary dietary source [62]. According to the World Health Organization, microelement deficiencies affect approximately 3 billion people worldwide, leading to severe health issues such as stunted growth, intellectual impairments, perinatal complications, and an increased risk of chronic diseases [63].

In the present study, Mn content varied between 18.73 mg/kg for L HELD 2025 and 51.40 mg/kg for L BEC 2024, with statistically significant differences detected across both genotypes and cultivation years. These results are higher than those obtained by Sinkovič et al. (2024) [4], which range from 11.44 to 30.14 mg/kg (Figure 4).

Regarding Cu content, values ranged from 7.29 mg/kg for line L HELD in 2024 to 23.42 mg/kg for the same genotype in the subsequent cultivation year. Notably, line L BEC proved to be the most stable genotype for Cu accumulation, exhibiting no significant inter-annual differences, whereas lines L HELD and L 301B showed an approximate threefold increase in Cu levels from 2024 to 2025.

Zinc (Zn) content varied from 25.56 mg/kg for line L HELD in 2024 to 52.27 mg/kg for line L 301B in 2025. An increase in Zn content from 2024 to 2025 was detected for lines L HELD and L 301B, whereas line L BEC demonstrated a decreasing trend (Figure 4).

These Zn values are consistent with the literature; Celmeli et al. (2018) [64] reported bean Zn contents between 17.81 and 37.90 mg/kg, a range that aligns with the performance of line L HELD, while Sinkovič et al. (2024) [4] observed a wider range of 20.15–90.27 mg/kg, which encompasses all recorded values.

Based on their respective mean values, the overall abundance pattern of the analyzed elements was established in the following descending order: K > Ca > Mg > Zn > Mn > Na > Cu. This identical elemental sequence was previously reported by Sinkovič et al. (2024) [4] for French bean pods.

Nutrient reference values (NRVs) for adults were evaluated per 100 g of fresh product in accordance with Regulation (EU) No. 1169/2011 [17] to assess the practical contribution of these genotypes to human dietary requirements. Consequently, line L BEC supplied the highest salt NRV percentage at 0.12%, followed by line L HELD with 0.08% and line L 301B with 0.06%. For magnesium, line L 301B achieved an NRV contribution of 15.69%, followed by line L HELD with 12.75% and line L BEC with 11,46%. In terms of potassium, line L 301B recorded the highest value, covering 39.22% of the NRV, followed by line L HELD with 22.90% and line L BEC with 14.96%. Calcium evaluation showed that line L 301B delivered the most efficient contribution at 16.20% of the NRV, followed by line L HELD with 11.04% and line L BEC with 6.54%.

When evaluating the microelement contributions, manganese values for line L 301B registered the highest NRV percentage at 22.37%, followed by line L BEC with 19.52% and line L HELD with 16.97%. For copper, line L 301B supplied 25.04% of the NRV, closely followed by line L HELD with 24.63% and line L BEC with 14.23%. The zinc NRV contribution followed a trend similar to that of manganese, with line L 301B reaching the highest value of 7.50%, followed by line L BEC with 4.90% and line L HELD with 4.75%.

In accordance with Regulation (EU) No 1169/2011 [17], it can be concluded that all analyzed genotypes provide significant amounts of Mg, K, Ca, Mn and Cu, thereby meeting the legal criteria to be considered major dietary sources of these essential minerals.

#### 3.1.8. Total Soluble Solids (TSS)

In the present study, the total soluble solids content (Table 1), expressed as °Brix, ranged from 4.20 ± 0.37% for line L 301B in 2024 to a maximum of 9.98 ± 0.43 for line L HELD in 2025. Statistically significant differences were observed across both genotypes and cultivation years. For the line L HELD, a dramatic and statistically significant increase was recorded, with °Brix values nearly doubling from 5.20 ± 0.29 in 2024 to 9.98 ± 0.43 in 2025. A similar increasing trend between the two consecutive years was detected for line L 301B, which rose significantly from 4.20 ± 0.37% to 7.84 ± 0.27. Conversely, line L BEC exhibited the highest stability for this parameter; although its values increased slightly from 4.48 ± 0.19 in 2024 to 5.33 ± 0.51 in 2025, this variation remained statistically non-significant, as both years shared the same significance letter. This synchronized surge in °Brix during the 2025 season for lines L HELD and L 301B closely mirrors the previously observed reduction in K and Ca contents. Under water deficit, plants can increase the concentration of osmotically active solutes, particularly soluble sugars, while inorganic ions such as K and Ca may also contribute to osmotic adjustment and maintenance of cellular turgor [65]. Our results are in agreement with Menga et al. (2023) [46] and Sinkovič et al. (2025) [66], who also found considerable variation in TSS between 1.97 and 11.33 °Brix. In contrast, L BEC showed the greatest stability in °Brix, with only a slight and non-significant increase between 2024 and 2025, indicating a relatively consistent total soluble solids content across cultivation years. This stability, together with its high total sugar content, suggests a consistent sweetness-related quality of the pods. In comparison, L HELD and L 301B showed marked increases in °Brix in 2025, reaching 9.98 ± 0.43 and 7.84 ± 0.27, respectively. These higher soluble-solids values may contribute to a sweeter sensory profile of the pods.

### 3.2. ISSR Characterization

**Figure 5:**
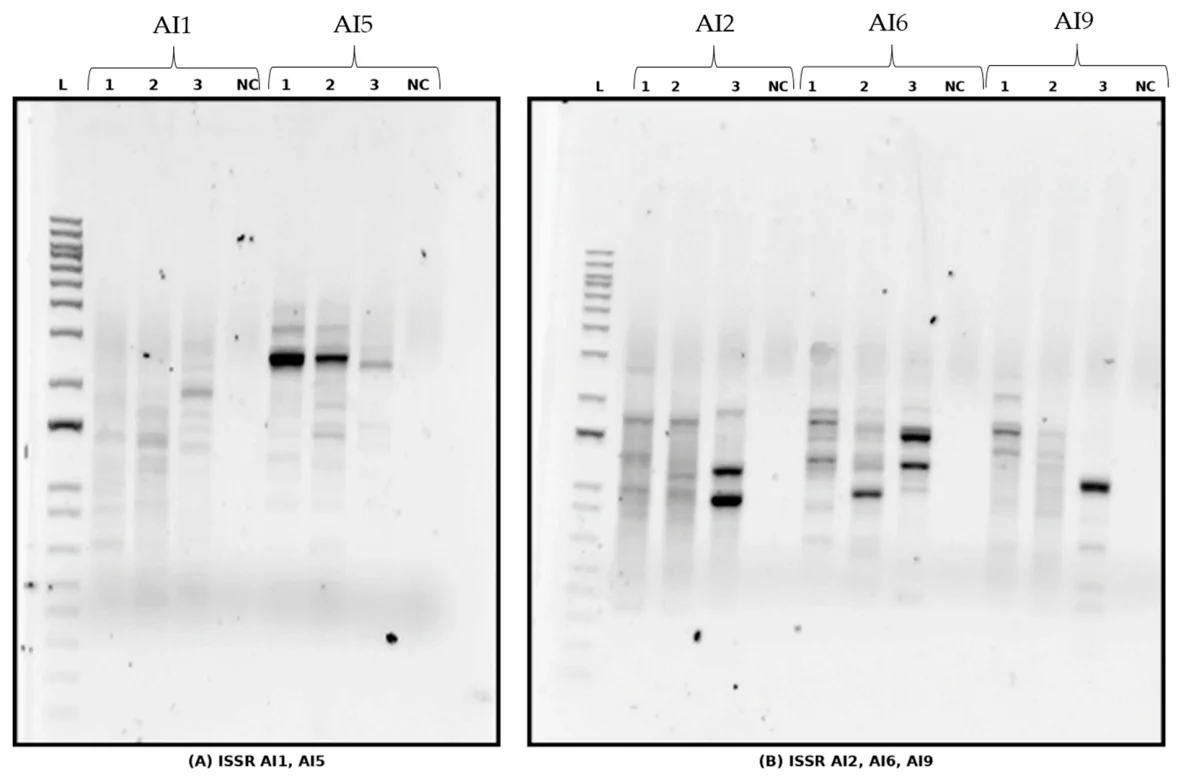
Electrophoretic profile resulting from the PCR reaction of three bean genotypes, where L: Ladder, 1: L301B, 2: L HELD, 3: L BEC, and NC: negative control. (**A**): AI1 and AI5 ISSR primers, and (**B**): AI2, AI6, AI9 ISSR primers.

The PCR products obtained by amplifying the genomic DNA of the three bean genotypes (L 301, L HELD, and L BEC) with the ISSR primers AI1, AI2, AI5, AI6, and AI9 exhibited well-defined bands. The patterns revealed polymorphism among the samples, with clear differences between the three genotypes. The negative controls for each primer showed no bands, confirming the absence of contamination. Based on the gel analysis, primers AI5, AI6, and AI9 were the most polymorphic and most useful for distinguishing the genotypes, whereas AI1 and especially AI2 showed a greater degree of similarity (less informative for discrimination, but useful as a reproducibility benchmark). A recurring pattern is the genetic closeness between L 301B and L HELD (most clearly reflected in shared bands at AI5, while AI2 shows general band conservation across all three genotypes), while L BEC repeatedly appears as the most divergent genotype, showing unique or absent bands relative to the other two at nearly all primers (AI1, AI5, AI6, AI9) (Figure 5).

ISSR analysis of the three bean lines (L 301B, L HELD, L BEC) using five primers (AI1, AI2, AI5, AI6, and AI9) yielded a total of 22 scorable bands, of which 12 (54.5%) were polymorphic (Table 4). Band scoring was compiled into a binary (1/0) presence/absence matrix, and genetic diversity statistics were calculated in RStudio. Nei’s gene diversity (He) and the Shannon-Wiener index (I) were calculated per band from band frequency across the three genotypes and averaged per primer, based on visual presence/absence (1/0) scoring of the ISSR gel images. AI9 was fully polymorphic (100% PPB) and AI5 highly informative (80% PPB), both showing the highest gene diversity and Shannon index values, whereas AI2 was completely monomorphic (0% PPB) across the three genotypes and therefore uninformative for differentiation, though useful as a reproducibility check. Overall, mean Nei’s gene diversity (He = 0.242) and Shannon index (I = 0.347) indicate a moderate level of genetic diversity among the three lines, with L 301B and L HELD showing the closest genetic similarity and L BEC emerging as the most genetically divergent line.

Table 4 summarizes the genetic diversity and polymorphism generated by each ISSR primer. A total of 22 bands were scored, of which 12 (54.5%) were polymorphic. AI9 and AI5 showed the highest polymorphism (100% and 80% PPB) and the highest diversity indices (He and Shannon I), while AI2 was fully monomorphic (0% PPB), confirming no discriminatory value for this primer among the three genotypes.

Pairwise genetic similarity analysis, calculated in RStudio, showed that L 301B and L HELD were the most closely related genotypes (Table 5), sharing 14 of their 22 scored bands and yielding the highest Jaccard (0.737) and Dice (0.848) coefficients, which corresponded to the smallest genetic distance (0.152). L 301B and L BEC showed an intermediate level of similarity (Jaccard = 0.600, Dice = 0.750; distance = 0.250), while L HELD and L BEC were the most genetically divergent pair, with the lowest similarity coefficients (Jaccard = 0.476, Dice = 0.645) and the largest genetic distance (0.355). This pattern is consistent with the primer-level results, in which L BEC repeatedly displayed unique or absent bands relative to the other two lines, confirming it as the most genetically distinct of the three bean genotypes, while L 301B and L HELD form a more closely related pair.

Table 5 presents the pairwise genetic similarity and distance among the three bean lines, based on Jaccard and Dice (Nei–Li) coefficients calculated from the ISSR presence/absence matrix. L 301B and L HELD showed the highest similarity (Jaccard = 0.737, Dice = 0.848), whereas L HELD and L BEC were the most genetically distant pair (Jaccard = 0.476, Dice = 0.645).

A high efficiency of ISSR markers in evaluating genetic diversity across 36 new lines and five common bean cultivars differentiating common beans was found by Sakhravi et al. (2023) [67], revealing strong molecular relationships and wide genetic diversity among the evaluated germplasm. Similarly, a substantial genetic variation among 50 common bean genotypes was identified [68].

### 3.3. Correlations

#### 3.3.1. Pearson Correlation

**Figure 6:**
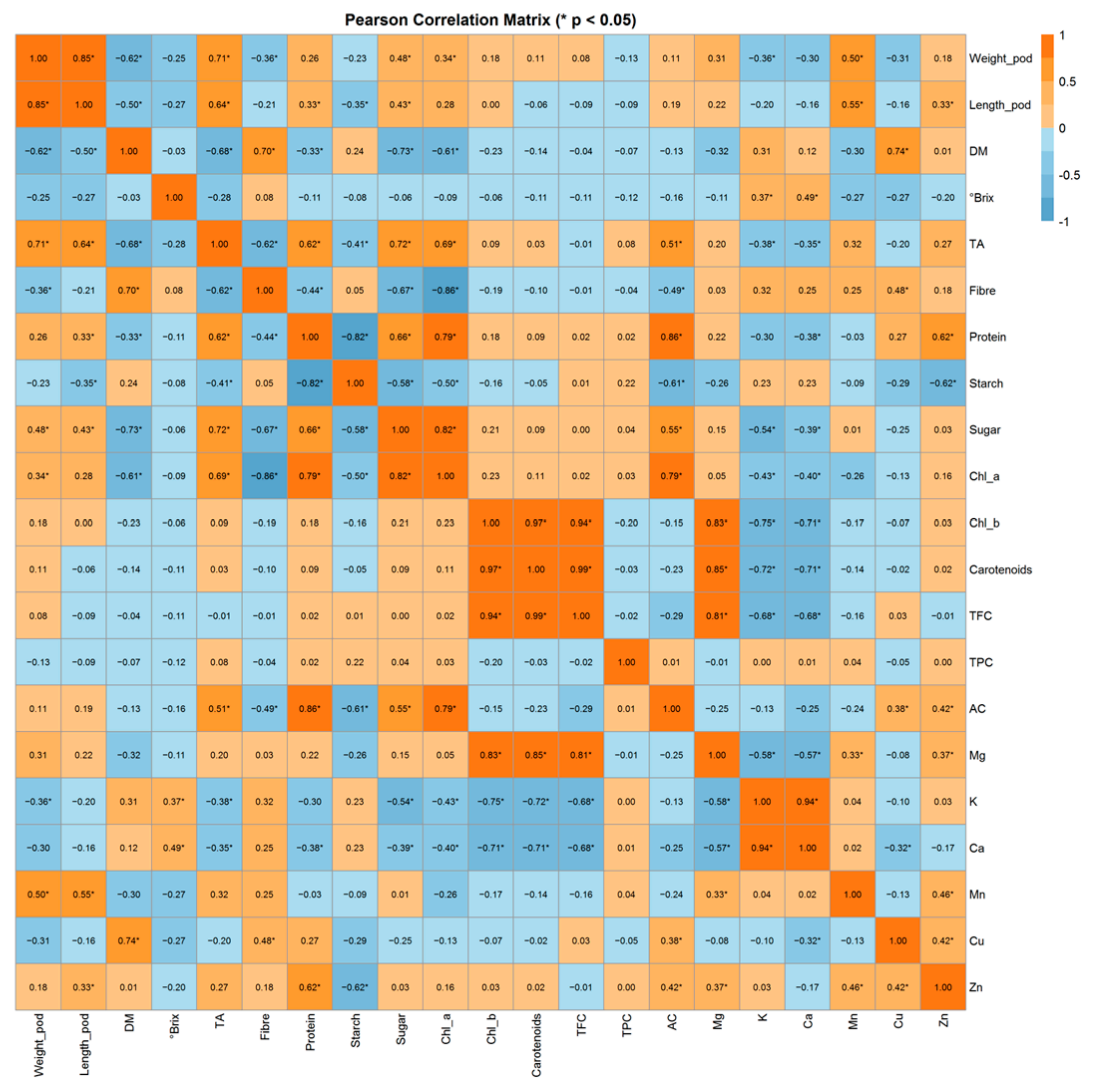
Color-coded Heatmap of the Pearson correlation matrix among 21 morphological, nutritional, and biochemical traits in bean pods across two experimental years. Pearson correlation coefficients for each pairwise comparison are shown in each cell. Positive correlations (from 0 to 1) are displayed in orange, negative correlations in blue (from 0 to −1). The color intensity (dark to light) indicates the strength and direction of the relationships between the traits. Asterisks (*) denote statistically significant pairwise correlations at *p* < 0.05.

The correlation analysis (Figure 6) revealed a moderate and statistically significant positive correlation between total sugars and antioxidant capacity (AC) (r = 0.55), as well as between °Brix and antioxidant capacity (r = 0.51).

Furthermore, a very strong positive correlation was observed between protein content and antioxidant capacity (r = 0.86). Similar results were reported by Márquez et al. (2024) [69], and Şener (2026) [70], who also found strong positive correlations among antioxidant capacity, total phenolics, flavonoids, and protein content.

Regarding protein accumulation, a moderate positive correlation was observed between protein and zinc content (r = 0.62). Zinc plays an essential role in gene regulation and expression, protein synthesis, nitrogen assimilation, carbohydrate metabolism, photosynthesis, phytohormone activity, seed production, and plant defense mechanisms against disease [71].

Titratable acidity (TA) displayed a strong positive association with pod weight (r = 0.71) and pod length (r = 0.64), suggesting that larger pods were associated with greater organic-acid accumulation. A positive correlation was also observed between TA and protein content (r = 0.62), as well as between TA and chlorophyll a (r = 0.69). These relationships suggest that pod size, acidity, protein concentration, and photosynthetic pigment content varied concomitantly among the studied samples.

A medium positive correlation was found between Mn and pod length (r = 0.55), as well as between Mn and pod weight (r = 0.50). Manganese is associated with numerous metabolic functions in plant cellular compartments and is an essential plant micronutrient for growth [72]. Mn also plays a role in the oxygen-evolving complex of photosystem II and cell wall formation [73]. Higher Mn availability supports vigorous growth, leading to larger and heavier pods.

Very strong correlations were also found for Mg-TFC (r = 0.81), Mg-Chl b (r = 0.83), Mg-carotenoids (r = 0.85), Chl_b-TFC (r = 0.94), and Chl_b-carotenoids (r = 0.97). The strong association between Mg and chlorophyll b may be related to the structural role of magnesium in chlorophyll molecules. These associations may reflect coordinated variation in magnesium nutrition, photosynthetic pigments, and secondary-metabolite accumulation, in agreement with Jin et al. [74], who reported that Mg supply influenced chlorophyll, photosynthetic performance, and flavonoid-related metabolites.

To further elucidate how these individual Pearson correlation patterns coordinate at a systemic level of each genotype across the two cultivation years, a Principal Component Analysis (PCA) was subsequently performed.

#### 3.3.2. Principal Component Analysis (PCA)

To provide a comprehensive view of how the evaluated genotypes behave across different growing conditions, the physical and biochemical datasets were subjected to Principal Component Analysis (PCA). This multivariate technique condenses the multidimensional data into a two-dimensional projection space, facilitating the direct visualization of inter-trait correlations and the identification of distinct trait associations within each line. As a result, the individual biplots generated for lines L 301B, L BEC, and L HELD offer a clear representation of their trait stability and dispersion across two consecutive cultivation years.

**Figure 7:**
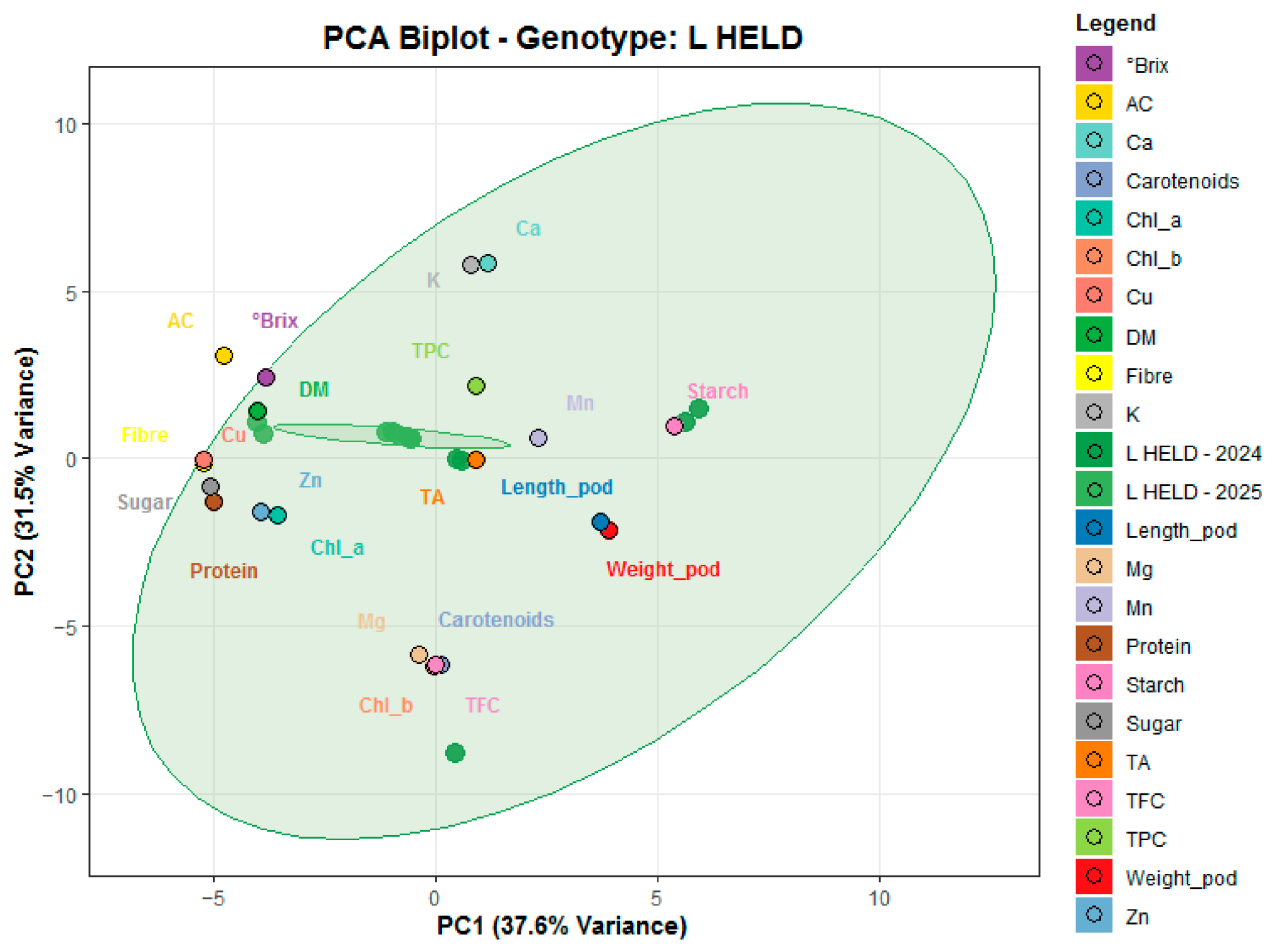
PCA correlation matrix for L HELD line.

The PCA biplot for genotype L HELD (Figure 7) characterizes tight clustering and restricted trait dispersion across years, with PC1 and PC2 describing a combined total variance of 69.1% (37.6% and 31.5%, respectively).

The geometric arrangement of the confidence ellipses reveals an asymmetrical pattern; the 2024 season occupies a large, widespread area encompassing multiple physiological parameters, whereas the 2025 season shrinks into an extremely narrow, strictly flattened horizontal cluster positioned closely along the PC1 axis.

**Figure 8:**
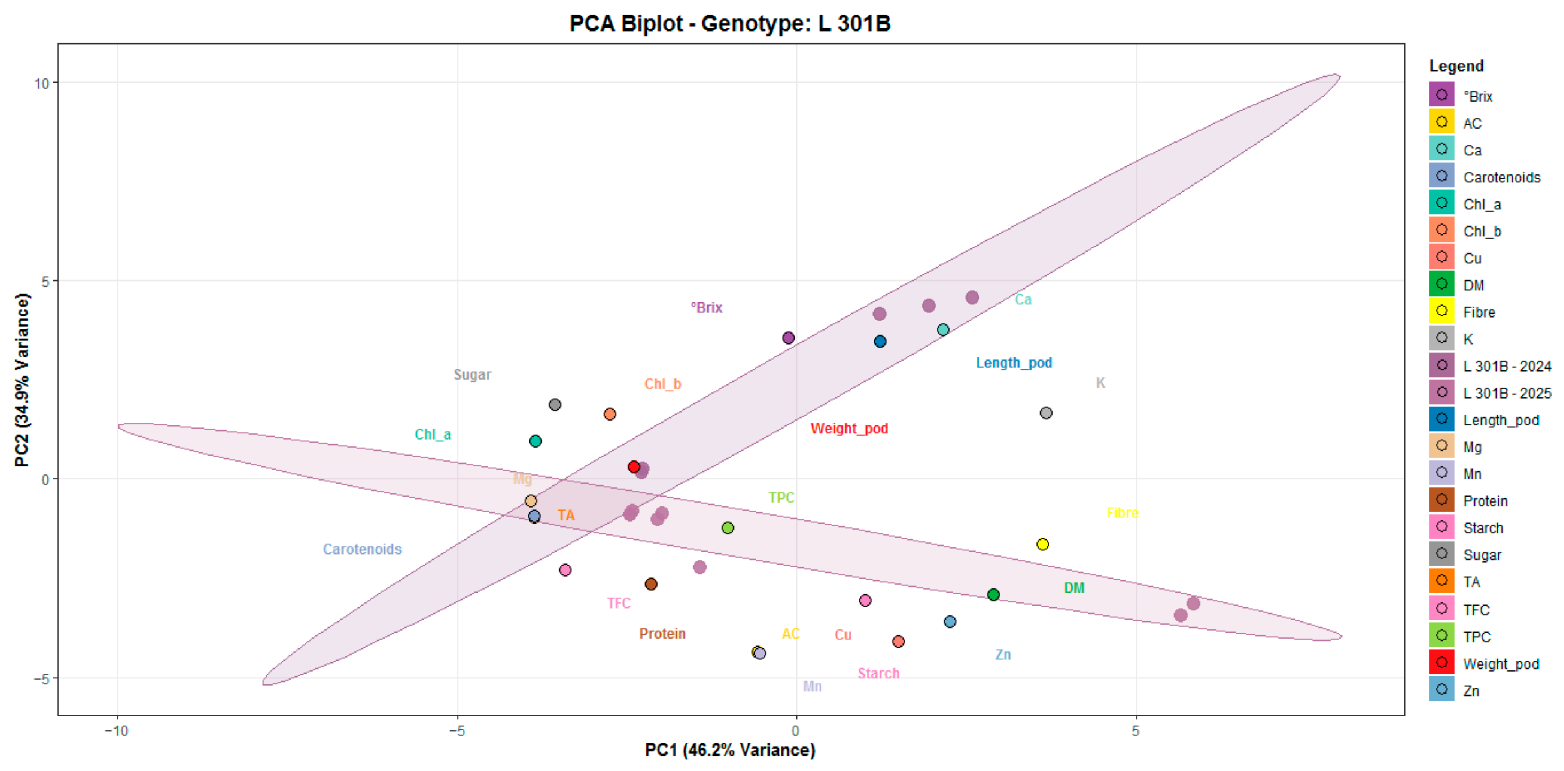
PCA correlation matrix for L 301B line.

PC1 tends to separate pod size from nutritional composition. Weight, length, and starch load strongly on the positive (right) side of PC1, whereas fiber, protein, sugar, and Cu load on the negative (left) side of PC1. Line L HELD showed an inverse relationship between physical yield/size parameters and nutritional density. Larger, heavier pods accumulate more starch and biomass, thereby lowering the concentrations of proteins, structural fibers, and trace minerals such as copper, as also highlighted in the Pearson correlation matrix (Figure 8). PC2 separates metabolic pathways involving mineral uptake and sugar accumulation from pathways governing pigment synthesis and secondary antioxidant metabolites. Mineral accumulation (Ca, K) and soluble solids (°Brix) load toward the top. Photosynthetic pigments (Chl_b) and bioactive antioxidants (TFC, carotenoids) load toward the bottom.

In 2024, line L HELD displayed a highly diverse metabolic baseline distributed across multiple quadrants. The samples from this normal season show strong associations with mineral clusters, including Ca, Mg, and Zn, as well as prominent structural and antioxidant traits like TPC, TFC, and carotenoids located in the lower quadrants.

In contrast, the 2025 cultivation year exhibited a narrower metabolic variation, with the samples clustering predominantly in the negative PC1 region, grouping tightly around °Brix, AC, and primary nutritional components such as protein, sugars, and Cu.

The PCA biplot for genotype L 301B (Figure 8) highlights a distinct structural and metabolic divergence between the two cultivation years, with PC1 and PC2 jointly accounting for a high total variance of 81.1% (46.2% and 34.9%, respectively).

The spatial distribution reveals an intersecting “X-shaped” orientation of the confidence ellipses, demonstrating that line L 301B switches between two distinct metabolic profiles. In 2024, the metabolic profile is strictly oriented toward structural development and mineral densification, positioning the samples along the negative PC2 axis. This profile is heavily characterized by maximum accumulations of Ca, Zn, ash, and structural elements like fiber and starch, which align with physical traits such as pod thickness. Conversely, in 2025, a complete metabolic shift occurs along a highly elongated ellipse stretching into the positive PC1 and PC2 quadrant. Interestingly, core parameters like pod weight, Chl b, and secondary metabolites like TFC, TPC, and carotenoids cluster tightly around the intersection of the two ellipses, confirming that line L 301B maintains a stable, robust homeostatic baseline for its key bioactive and commercial traits.

**Figure 9:**
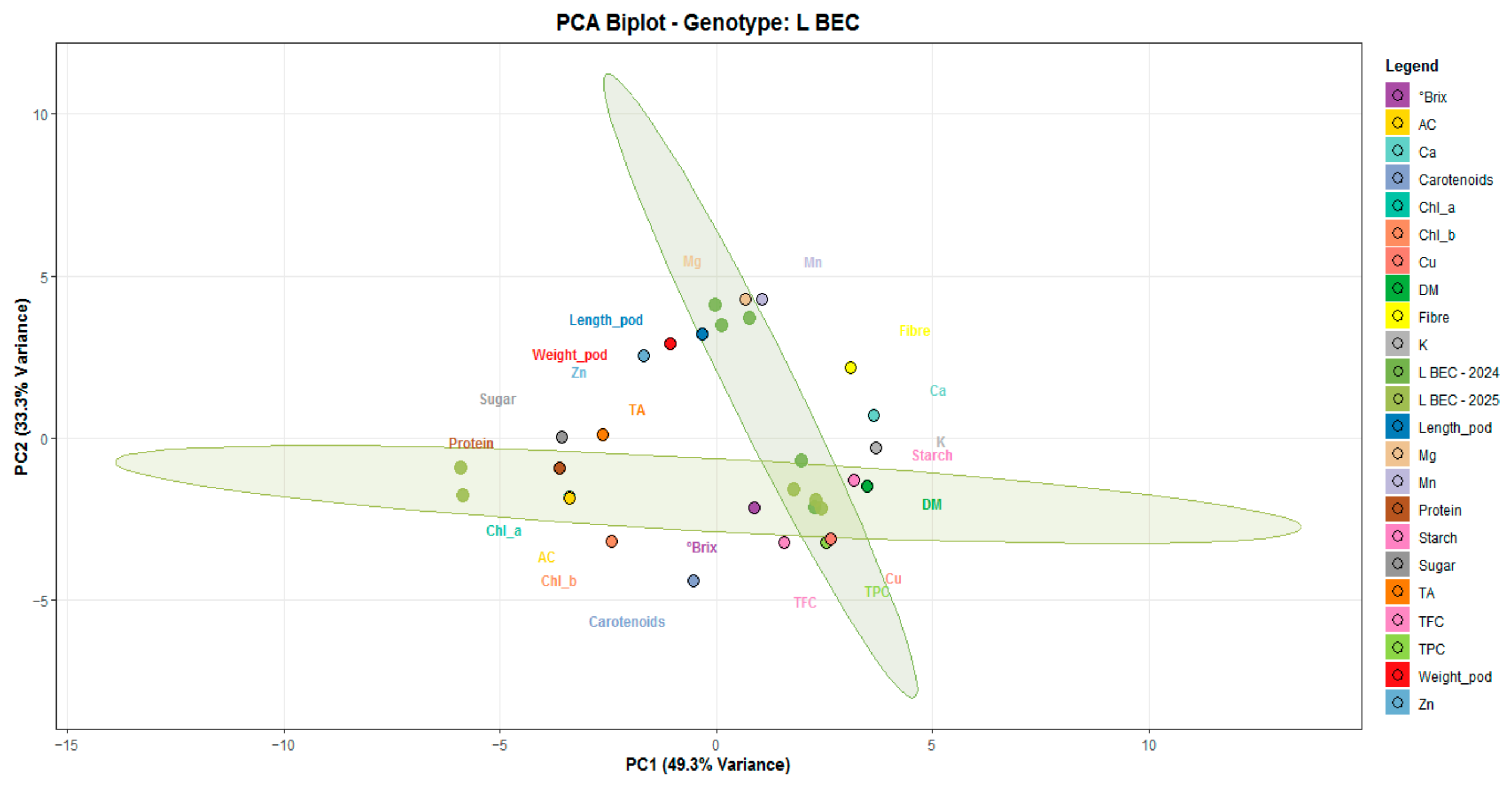
PCA correlation matrix for L BEC line.

The PCA biplot for genotype L BEC (Figure 9) reveals a profound and highly dynamic macro-metabolic reconfiguration between the two cultivation seasons, with PC1 and PC2 accounting for an excellent cumulative variance of 82.6% (49.3% and 33.3%, respectively).

The spatial projection is characterized by two strictly perpendicular confidence ellipses, validating a total directional shift in the metabolic machinery. In the 2024 season, the genotype activates a strictly horizontal, storage- and biomass-accumulation aligned along the PC1 axis. This phenotypic response is heavily driven by the synthesis of structural carbohydrates and basic nutrition, positioning the samples in close proximity to vectors for protein, total sugars, starch, and DM.

Conversely, the 2025 season triggers a complete metabolic pivot, forcing a vertical elongation of the confidence ellipse along the PC2 axis. The 2025 metabolic profile is strongly pulled by physical development and mineral mobilization, correlating intensely with maximum pod elongation, pod weight, and an elevated accumulation of trace minerals such as Mg, Mn, and Zn.

## 4. Study Limitations and Future Directions

Although this study provides valuable insights into the multi-trait profiles of the new bean genotypes, several limitations should be acknowledged to contextualize the findings and guide future research.

First, the study was conducted over only two consecutive cultivation years, providing a preliminary assessment of the agronomic, nutritional, and biochemical performance of the investigated genotypes. Although year-to-year variations were observed for several traits, the experimental design did not enable the identification of the specific environmental factors underlying these differences. Therefore, multi-year and multi-condition cultivation trials are required to evaluate the stability and adaptability of genotype performance across diverse growing conditions.

Second, the effects of cooking processing on nutritional quality were not investigated. Consequently, their influence on the retention, stability, and bio accessibility of vitamins, pigments, proteins, and other bioactive constituents remains to be elucidated.

Third, antinutritional factors, including phytic acid, tannins, lectins, saponins, and oxalates, were not quantified. As these compounds can affect nutrient utilization and mineral bioavailability, their evaluation would provide a more comprehensive assessment of the nutritional value of the investigated genotypes.

Fourth, genetic characterization relied exclusively on ISSR molecular markers. While these markers effectively discriminated among the genotypes, they do not provide the genomic resolution afforded by SNP-based approaches or next-generation sequencing technologies, which could facilitate the identification of genomic regions and candidate genes associated with key agronomic and nutritional traits.

Finally, the evaluation was restricted to three elite breeding lines, limiting the extent to which the observed metabolic relationships and Pearson correlations can be generalized across the broader *Phaseolus vulgaris* L. germplasm. Further validation using larger and more genetically diverse populations would strengthen the robustness and applicability of the findings.

## 5. Conclusions

The comprehensive integration of morphological, biochemical, multi-elemental, and molecular analyses in this study successfully characterized three novel common bean (*Phaseolus vulgaris* L.) genotypes, demonstrating highly distinct physiological profiles and specialization pathways:

- L BEC genotype excelled in commercial and biometric attributes, recording the highest pod weight and length. Biochemically, it achieved the highest concentrations of total protein, total sugars, titratable acidity, and assimilatory pigments. Crucially, L BEC displayed a dominant antioxidant profile, driven by its exceptional total phenolic content and radical scavenging capacity, standing out with the highest salt (NaCl) equivalent, although still extremely low (0.12% NRV). It ranks second or third for most minerals but maintains balanced levels of Mg, Mn, and Zn. These findings optimize L BEC for high-value functional foods and nutraceutical applications.
- L 301B purple-podded genotype specialized in the accumulation of structural and cellular protectants, yielding the highest overall dry matter, ash, and total fiber. Benefiting from a targeted upregulation of the anthocyanin biosynthetic pathway, L 301B achieved superior total flavonoid accumulation. Furthermore, it demonstrated the most efficient macroelement biofortification, showing the highest NRV contribution for almost all minerals: Mg, K, Ca, Mn, Cu, and Zn. Its values are consistently above the other genotypes, with particularly strong contributions for potassium (39.22% NRV), calcium (16.20% NRV), magnesium (15.69% NRV), and manganese (22.37% NRV). Characterized by strict inter-annual stability across pod weight, ash, fiber, and mineral accumulation, this line is ideal for mineral-fortified processing and targeted breeding programs.
- L HELD genotype was characterized by the highest content of starch (36.16%) and intermediate NRVs across all minerals. It does not rank first for any element but consistently occupies second place for Mg, K, Ca, and Cu. L HELD with a maximum of 9.98 °Brix could enhance pod palatability and natural sweetness. This profile renders L HELD highly suitable for energy-dense formulations and starch-rich processed foods.

At the systemic level, Pearson correlation analysis revealed strong associations among nutritional components, photosynthetic pigments, antioxidant capacity, and mineral composition. A strong negative correlation between total sugars and dry matter indicated that higher sugar content was associated with lower dry matter content in the analyzed samples.

Structurally, these functional divergences were strongly corroborated by molecular data. ISSR fingerprinting yielded 22 scorable bands with 54.5% polymorphism, reflecting a moderate level of genetic diversity (mean He = 0.242; I = 0.347). Pairwise similarity matrix calculations established a close genetic affinity between L 301B and L HELD, which shared 14 bands and exhibited the highest Jaccard (0.737) and Dice (0.848) coefficients (minimum genetic distance of 0.152). Conversely, molecular metrics isolated L BEC as the most genetically divergent genotype, displaying maximum genetic distance when paired with L HELD (0.355), primarily driven by highly informative polymorphic patterns at primers AI9 and AI5.

Ultimately, mapping this internal biodiversity under seasonal conditions is essential for securing long-term food security and building resilient food chains. Correlating genetic and mineral fingerprinting with multi-trait phenotypic profiling delivers a robust, high-resolution toolkit for accelerated marker-assisted breeding selection, commercial cultivar authentication, and advanced food safety monitoring.

## Figures and Tables

**Table 1:** ISSR primer sequences and annealing temperature.

Primer Code	ISSR Primer	Primer Sequence (5′3′)	Standard Annealing Temperature (Tm)
AI1	818	5′-CACACACACACACACAG-3′	Tm = 52.1
AI2	827	5′-ACACACACACACACACG-3′	Tm = 54.9
AI5	848	5′-CACACACACACACACARG-3′	Tm = 50.6
AI6	855	5′-ACACACACACACACACYT-3′	Tm = 60.2
AI9	ISSR9	5′-ATGATGATGATGATGATG-3′	Tm = 51.3

**Table 2:** Morphological and quality parameters of bean genotypes.

Genotype-Year	Weigh/ Pod (g)	Length/ Pod (cm)	DM %	°Brix	TA (Equiv. Citric Acid g/100 g)	Ash %	Total Fiber %	Protein %	Starch %	Total Sugar %
L HELD-2024	19.55 ± 0.97 c	22.30 ± 1.82 c	12.37 ± 0.08 b	5.20 ± 0.29 c	1.01 ± 0.02 c	4.91 ± 0.05 e	11.30 ± 0.75 b	26.33 ± 0.04 d	43.51 ± 1.83 a	11.03 ± 0.77 c
L HELD-2025	3.84 ± 0.41 d	11.06 ± 1.04 d	17.19 ± 0.12 a	9.98 ± 0.43 a	0.87 ± 0.03 c	6.84 ± 0.01 a	14.82 ± 0.37 a	31.54 ± 0.33 b	28.80 ± 0.18 cd	12.69 ± 0.15 bc
L 301B-2024	7.66 ± 0.89 d	16.60 ± 0.91 cd	12.47 ± 0.13 b	4.20 ± 0.37 d	0.87 ± 0.05 c	6.47 ± 0.04 b	13.62 ± 0.33 a	29.95 ± 0.33 c	30.77 ± 0.16 bc	11.15 ± 0.07 c
L 301B-2025	5.03 ± 0.48 d	11.69 ± 1.10 d	17.95 ± 1.07 a	7.84 ± 0.27 b	0.76 ± 0.04 c	6.51 ± 0.01 b	15.47 ± 0.30 a	31.01 ± 0.22 bc	34.34 ± 0.04 b	6.76 ± 0.29 d
L BEC-2024	32.52 ± 2.87 a	82.88 ± 8.60 a	11.04 ± 0.13 c	4.48 ± 0.19 d	1.50 ± 0.12 b	5.94 ± 0.01 d	14.73 ± 0.53 a	31.97 ± 0.41 b	26.54 ± 0.14 d	14.25 ± 0.70 b
L BEC-2025	27.80 ± 2.72 b	59.00 ± 4.64 b	9.20 ± 0.04 d	5.33 ± 0.51 c	1.92 ± 0.19 a	6.20 ± 0.11 c	7.98 ± 0.90 c	37.50 ± 0.21 a	24.81 ± 1.82 d	17.40 ± 1.06 a

Data are presented as Mean ± Standard Deviation (*n* = 3). Means within the same column followed by different letters differ significantly according to Tukey’s HSD test (*p* < 0.05). Comparisons were performed independently for each parameter.

**Table 3:** Total phenolic, total flavonoid content, and antioxidant capacity.

Genotype–Year	TPC (mg GAE/100 g DW)	TFC (mg RE/100 g DW)	AC (mg TE/100 g DW)
L Held-2024	463.977 ± 9.353 c	213.500 ± 13.169 b	5735.730 ± 294.791 e
L Held-2025	440.457 ± 26.904 c	139.190 ± 12.303 c	23,489.089 ± 1391.275 b
L 301B-2024	885.087 ± 45.390 b	223.009 ± 9.881 b	10,989.838 ± 95.893 d
L 301B-2025	606.261 ± 44.380 c	294.522 ± 9.029 a	18,603.853 ± 1273.574 c
L BEC-2024	1290.623 ± 116.182 a	103.178 ± 1.604 c	13,590.811 ± 730.810 d
L BEC-2025	1280.100 ± 79.994 a	325.709 ± 6.898 a	41,590.988 ± 1501.102 a

Data are presented as Mean ± Standard Deviation (*n* = 3). Means within the same column followed by different letters differ significantly according to Tukey’s HSD test (*p* < 0.05). Comparisons were performed independently for each parameter.

**Table 4:** ISSR Genetic diversity and polymorphism.

Primer	Total Bands	Polymorphic Bands	Polymorphism (PPB %)	Nei’s Gene Diversity (He)	Shannon Index (I)
AI1	5	2	40.0%	0.178	0.255
AI5	5	4	80.0%	0.356	0.509
AI2	4	0	0.0%	0.000	0.000
AI6	5	3	60.0%	0.267	0.382
AI9	3	3	100.0%	0.444	0.637
Total/mean	22	12	54.5%	0.242	0.347

Notes: PPB (Percentage of Polymorphic Bands) = number of polymorphic bands/total number of bands scored for that primer.

**Table 5:** Pairwise genetic similarity between genotypes.

Genotype Pair	Jaccard Similarity	Dice (Nei–Li) Similarity	Genetic Distance (1-Dice)
L 301B vs. L HELD	0.737	0.848	0.152
L 301B vs. L BEC	0.600	0.750	0.250
L HELD vs. L BEC	0.476	0.645	0.355

Notes: Jaccard similarity = a/(a + b + c); Dice (Nei–Li) similarity = 2a/(2a + b + c), where a = number of bands shared by both genotypes, and b, c = bands unique to each genotype. Genetic distance was calculated as 1-Dice.

## Data Availability

The original contributions presented in this study are included in the article. Further inquiries can be directed to the corresponding author.

## References

[1] Food and Agriculture Organization of the United Nations FAOSTAT. https://www.fao.org/faostat/en/#data/QCL.

[2] CBI (Centre for the Promotion of Imports from Developing Countries) The European Market Potential for Green Beans. https://www.cbi.eu/market-information/fresh-fruit-vegetables/green-beans.

[3] Carović-Stanko K., Maloić M., Pintar J., Liber Z., Radosavljević I., Bedeković D., Guberac S., Očić V., Lazarević B. (2018). Nutritional quality of phaseolin types and morphotypes of green bean (*Phaseolus vulgaris* L.). Agrociencia.

[4] Sinkovič L., Blažica V., Blažica B., Meglič V., Pipan B. (2024). How Nutritious Are French Beans (*Phaseolus vulgaris* L.) from the Citizen Science Experiment?. Plants.

[5] Aquino-Bolaños E.N., Garzón-García A.K., Alba-Jiménez J.E., Chávez-Servia J.L., Vera-Guzmán A.M., Carrillo-Rodríguez J.C., Santos-Basurto M.A. (2021). Physicochemical Characterization and Functional Potential of *Phaseolus vulgaris* L. and *Phaseolus coccineus* L. Landrace Green Beans. Agronomy.

[6] Carbas B., Machado N., Oppolzer D., Ferreira L., Queiroz M., Brites C., Rosa E.A., Barros A.I. (2020). Nutrients, Antinutrients, Phenolic Composition, and Antioxidant Activity of Common Bean Cultivars and Their Potential for Food Applications. Antioxidants.

[7] Chávez-Mendoza C., Sánchez E. (2023). Antioxidant capacity and nutraceutical compounds content of six common bean (*Phaseolus vulgaris* L.) varieties harvested in Morelos, Mexico. Not. Sci. Biol..

[8] Espinosa-Alonso L.G., Gálvez-Morales J.D.J., Udenigwe C.C., Martinez-Ayala A.L., Valdez-Morales M., Valdez-Ortiz A., Medina-Godoy S. (2025). Chemical and nutraceutical characterization of coproducts obtained during the protein concentrate preparation from azufrado common bean grains. Biocatal. Agric. Biotechnol..

[9] Bernardi C., Finetti F., Trabalzini L. (2026). The Nutraceutical Promise of *Phaseolus vulgaris* L.: Bioactive Compounds for Health Promotion and Prevention of Chronic Noncommunicable Diseases. Biology.

[10] Oli P., Punetha S., Dev R. (2026). Agro-morphological and biochemical characterization of *Phaseolus vulgaris* landraces from the Indian Western Himalayas. Food Hum..

[11] Vieler L., Födisch J., Paquignon T., Schmidt M.A., Acir I.-H., Etzbach L., Schweiggert-Weisz U. (2026). Comparative evaluation of common bean (*Phaseolus vulgaris* L.) varieties as raw material for protein ingredient production. J. Agric. Food Res..

[12] Smeriglio A., Imbesi M., Ingegneri M., Rando R., Mandrone M., Chiocchio I., Poli F., Trombetta D. (2025). From Waste to Resource: Nutritional and Functional Potential of Borlotto Bean Pods (*Phaseolus vulgaris* L.). Antioxidants.

[13] Mu Q., Zhang T., Zhang Y., Feng G., Yan Z., Yang X., Liu D., Liu C. (2026). Research on the molecular mechanism of fiber formation in the suture of snap bean (*Phaseolus vulgaris* L.) pods. Plant Physiol. Biochem..

[14] Jan S., Rather I.A., Sofi P.A., Wani M.A., Sheikh F.A., Bhat M.A., Mir R.R. (2021). Characterization of common bean (*Phaseolus vulgaris* L.) germplasm for morphological and seed nutrient traits from Western Himalayas. Legume Sci..

[15] Yan Y., Khalid W., Fan M., Qian H., Li Y., Wang L. (2026). Kidney Beans (*Phaseolus vulgaris* L.): Composition, Health Benefits, and Health-Oriented Processing Strategies. Compr. Rev. Food Sci. Food Saf..

[16] El Sheikha A.F., Allam A.Y., Taha M., Varzakas T. (2022). How Does the Addition of Biostimulants Affect the Growth, Yield, and Quality Parameters of the Snap Bean (*Phaseolus vulgaris* L.)? How Is This Reflected in Its Nutritional Value?. Appl. Sci..

[17] European Parliament (2011). Regulation (EU) No 1169/2011 of the European Parliament and of the Council of 25 October 2011 on the Provision of Food Information to Consumers.

[18] Fan G., Beta T. (2017). Discrimination of geographical origin of Napirira bean (*Phaseolus vulgaris* L.) based on phenolic profiles and antioxidant activity. J. Food Compos. Anal..

[19] Yang Q.-Q., Farha A.K., Cheng L., Kim G., Zhang T., Corke H. (2020). Phenolic content and in vitro antioxidant activity in common beans (*Phaseolus vulgaris* L.) are not directly related to anti-proliferative activity. Food Biosci..

[20] Neupane B.S., Olee D., Shrestha D.S., Kharel G.P., Koirala N. (2024). Nutritional and phytochemicals analysis of high-altitude common bean (*Phaseolus vulgaris* L.) cultivars of Nepal. eFood.

[21] Taïbi K., Taïbi F., Ait Abderrahim L., Ennajah A., Belkhodja M., Mulet J.M. (2016). Effect of salt stress on growth, chlorophyll content, lipid peroxidation and antioxidant defence systems in *Phaseolus vulgaris* L.. S. Afr. J. Bot..

[22] Hasnat H., Shompa S.A., Islam M.M., Alam S., Richi F.T., Emon N.U., Ashrafi S., Ahmed N.U., Chowdhury M.N.R., Fatema N. (2024). Flavonoids: A treasure house of prospective pharmacological potentials. Heliyon.

[23] Aryal S., Baniya M.K., Danekhu K., Kunwar P., Gurung R., Koirala N. (2019). Total Phenolic Content, Flavonoid Content and Antioxidant Potential of Wild Vegetables from Western Nepal. Plants.

[24] Valdés S.T., Coelho C.M.M., Michelluti D.J., Tramonte V.L.C.G. (2011). Association of genotype and preparation methods on the antioxidant activity and antinutrients in common beans (*Phaseolus vulgaris* L.). LWT–Food Sci. Technol..

[25] Sparvoli F., Giofré S., Cominelli E., Avite E., Giuberti G., Luongo D., Gatti E., Cianciabella M., Daniele G.M., Rossi M. (2021). Sensory Characteristics and Nutritional Quality of Food Products Made with a Biofortified and Lectin-Free Common Bean (*Phaseolus vulgaris* L.) Flour. Nutrients.

[26] Contreras Á., Carrasco C., Concha-Meyer A., Plaza A., Alarcón M., González I. (2024). Role of processing and encapsulation in the protection and bioaccessibility of phenolic compounds from Chilean Tórtola common bean (*Phaseolus vulgaris* L.). LWT.

[27] Tuseef M., Lela L., Carlucci V., Russo D., Santopietro F., Mangieri C., Faraone I., Benedetto N., Brancaleone V., Milella L. (2026). From waste to value: A systematic review of phytochemicals and functional properties of common bean (*Phaseolus vulgaris* L.) by-products. Food Chem..

[28] Dobrin A., Nedeluș A., Bujor O., Moț A., Zugravu M., Bădulescu L. (2019). Nutritional quality parameters of fresh red tomato varieties cultivated in organic system. Sci. Pap. Ser. B Hortic..

[29] European Commission (2014). Commission Implementing Regulation (EU) No 974/2014 of 11 September 2014 Laying Down the Refractometry Method of Measuring Dry Soluble Residue in Products Processed from Fruit and Vegetables.

[30] (2003). Fruit and Vegetable Products—Determination of Soluble Solids—Refractometric Method.

[31] Lagunovschi-Luchian V., Dobrin A., Tatu O., Bădulescu L. (2019). Research on the influence of modern culture technologies on morphometric and biochemical characterizations of eggplant varieties. Sci. Pap. Ser. B Hortic..

[32] (1998). Fruit and Vegetable Products—Determination of Titratable Acidity.

[33] Livadariu O., Constantin C.G., Boiu-Sicuia O.-A., Dobrin A., Ion V.A. (2024). Assessment of Aseptic and Non-Aseptic Systems’ Influence on Basil (*Ocimum basilicum* L.) Microplants. Plants.

[34] Ion V.A., Nicolau F., Petre A., Bujor O.C., Bădulescu L. (2020). Variation of bioactive compounds in organic *Ocimum basilicum* L. during freeze-drying processing. Sci. Pap. Ser. B Hortic..

[35] Constantin C.G., Zugravu M.M., Georgescu M., Constantin M.F., Moț A., Paraschiv M., Dobrin A. (2023). The Impact of the Growing Substrate on Morphological and Biochemical Features of *Salicornia europaea* L.. Appl. Sci..

[36] Bădulescu L., Bujor-Nenița O.C., Dobrin A., Stan A., Zugravu M., Ion V.A. (2019). Bioactive compounds and quality parameters in different organic apple varieties and their natural value-added powders. Fruit. Grow. Res..

[37] Moț A., Madjar R., Bădulescu L., Dobrin A., Constantin C.G., Mihalache M. (2021). Heavy metal uptake patterns by spinach (*Spinacia oleracea* L.) grown on contaminated different soil types. Sci. Pap. Ser. A Agron..

[38] Wafula E.N., Wainaina I.N., Buvé C., Nguyen N.-D.-T., Kinyanjui P.K., Saeys W., Sila D.N., Hendrickx M. (2020). Application of near-infrared spectroscopy to predict the cooking times of aged common beans (*Phaseolus vulgaris* L.). J. Food Eng..

[39] Kul R., Yıldırım E. (2023). Evaluation of Some Agro-morphological Characteristics of Dwarf Snap Bean Genotypes Collected from Erzurum Province. Turk. J. Agric. Food Sci. Technol..

[40] Bhattacharjee U., Uppaluri R.V.S. (2023). Growth and nutritional characteristics of *Phaseolus vulgaris* and Jeevamrutha bio-fertilizer-vermicompost system. Bioresour. Technol. Rep..

[41] García-Fernández C., Jurado M., Campa A., Bitocchi E., Papa R., Ferreira J.J. (2023). Genetic control of pod morphological traits and pod edibility in a common bean RIL population. Theor. Appl. Genet..

[42] Karavidas I., Ntatsi G., Marka S., Ntanasi T., Consentino B.B., Sabatino L., Iannetta P.P.M., Savvas D. (2023). Fresh Pod Yield, Physical and Nutritional Quality Attributes of Common Bean as Influenced by Conventional or Organic Farming Practices. Plants.

[43] Polania J.A., Poschenrieder C., Beebe S., Rao I.M. (2016). Effective Use of Water and Increased Dry Matter Partitioned to Grain Contribute to Yield of Common Bean Improved for Drought Resistance. Front. Plant Sci..

[44] Shi Y., Pu D., Zhou X., Zhang Y. (2022). Recent progress in the study of taste characteristics and the nutrition and health properties of organic acids in foods. Foods.

[45] Akçura M., Müftüoğlu N.M., Kaplan M., Türkmen C. (2020). Nutrient Potential and Mineral Contents of Some Vegetable Cluster Bean Genotypes. Cereal Chem..

[46] Menga V., Fares C., Campa A., Ferreira J.J., Bitocchi E., Papa R., Beleggia R. (2023). Variability of Nutritional, Antioxidant, and Textural Traits of a Collection of Snap Beans of Different Colors. Horticulturae.

[47] Gartner W., Nienhuis J., Bethke P.C., Kisha T.J. (2018). Variation Among Edible Podded Snap Bean Accessions for Pod and Seed Sugar Content. Bean Impr. Coop. Annu. Rep..

[48] Hu B., Zhu J., Wu H., Xu K., Zhai H., Guo N., Gao Y., Yang J., Zhu D., Xia Z. (2020). Enhanced Chlorophyll Degradation Triggers the Pod Degreening of “Golden Hook,” a Special Ecotype in Common Bean (*Phaseolus vulgaris* L.). Front. Genet..

[49] Cubas C., Lobo M.G., González M. (2008). Optimization of the extraction of chlorophylls in green beans (*Phaseolus vulgaris* L.) by N,N-dimethylformamide using response surface methodology. J. Food Compos. Anal..

[50] Huang P.-H., Cheng Y.-T., Chan Y.-J., Lu W.-C., Li P.-H. (2022). Effect of Heat Treatment on Nutritional and Chromatic Properties of Mung Bean (*Vigna radiata* L.). Agronomy.

[51] Martins T., Barros A.N., Rosa E., Antunes L. (2023). Enhancing Health Benefits through Chlorophylls and Chlorophyll-Rich Agro-Food: A Comprehensive Review. Molecules.

[52] Manoj N.S., O’Maoileidigh D.S. (2026). Activity, contribution, and regulation of seed pod photosynthesis. Discov. Life.

[53] Gómez-Martín C., Capel C., González A.M., Lebrón R., Yuste-Lisbona F.J., Hackenberg M., Oliver J.L., Santalla M., Lozano R. (2020). Transcriptional Dynamics and Candidate Genes Involved in Pod Maturation of Common Bean (*Phaseolus vulgaris* L.). Plants.

[54] Belmont R., Bernal L., Padilla-Chacón D., Coello P., Martínez-Barajas E. (2022). Starch accumulation in bean fruit pericarp is mediated by the differentiation of chloroplasts into amyloplasts. Plant Sci..

[55] Gorbe E., Morard M., Rausell-Moreno A., Calatayud Á., Penella C. (2025). Exploring nutritional quality and bioactive compounds in Mediterranean bean landraces. J. Food Compos. Anal..

[56] Kan L., Nie S., Hu J., Wang S., Cui S.W., Li Y., Xu S., Wu Y., Wang J., Bai Z. (2017). Nutrients, phytochemicals and antioxidant activities of 26 kidney bean cultivars. Food Chem. Toxicol..

[57] Nina N., Theoduloz C., Paillán H., Jiménez-Aspee F., Márquez K., Schuster K., Becker L., Oellig C., Frank J., Schmeda-Hirschmann G. (2023). Chemical profile and bioactivity of Chilean bean landraces (*Phaseolus vulgaris* L.). J. Funct. Foods.

[58] Celebioglu B., Hart J.P., Porch T., Griffiths P., Myers J.R. (2023). Genome-Wide Association Study to Identify Possible Candidate Genes of Snap Bean Leaf and Pod Color. Genes.

[59] Ali A.A.H. (2023). Overview of the vital roles of macro minerals in the human body. J. Trace Elem. Miner..

[60] Nieder R., Benbi D.K., Reichl F.X. (2018). Microelements and Their Role in Human Health. Soil Components and Human Health.

[61] Tränkner M., Tavakol E., Jákli B. (2018). Functioning of Potassium and Magnesium in Photosynthesis, Photosynthate Translocation and Photoprotection. Physiol. Plant..

[62] Cahyono J., Soetarejo F.E., Ismadji S. (2023). Utilization of sodium alginate hydrogel as a sustainable planting medium. AgroLife Sci. J..

[63] Galić L., Vukadinović V., Nikolin I., Lončarić Z. (2025). Soil Properties and Microelement Availability in Crops for Human Health: An Overview. Crops.

[64] Celmeli T., Sari H., Canci H., Sari D., Adak A., Eker T., Toker C. (2018). The Nutritional Content of Common Bean (*Phaseolus vulgaris* L.) Landraces in Comparison to Modern Varieties. Agronomy.

[65] Turner N.C. (2017). Turgor maintenance by osmotic adjustment, an adaptive mechanism for coping with plant water deficits. Plant Cell Environ..

[66] Sinkovič L., Hasanaklou H.T., Neji M., Plestenjak E., Dolničar P., Meglič V., Pipan B. (2025). Combining multi-criteria decision analysis with agro-morphological-biochemical-molecular traits of interest for use in breeding in promising common bean breeding lines (*Phaseolus vulgaris* L.). Cogent Food Agric..

[67] Sakhravi A., Dehdari M., Amiri Fahliani R. (2023). Genetic relationships among common bean (*Phaseolus vulgaris* L.) genotypes using ISSR markers. Gene Rep..

[68] Chhetri A.B., Lal J.P., Nair R.M., Singh A.K. (2025). Genetic diversity for pod and seed traits in common bean (*Phaseolus vulgaris* L.). Discov. Plants.

[69] Márquez K., Arriagada O., Pérez-Díaz R., Cabeza R.A., Plaza A., Arévalo B., Meisel L.A., Ojeda D., Silva H., Schwember A.R. (2024). Nutritional Characterization of Chilean Landraces of Common Bean. Plants.

[70] Şener A. (2026). Effects of nitrogen top-dressing treatments and development stages on morphological, colorimetric and biochemical characteristics of fresh bean pods (*Phaseolus vulgaris* L.). Sci. Rep..

[71] Kachinski W.D., Ávila F.W., Muller M.M.L., Reis A.R., Rampim L., Vidigal J.C.B. (2020). Nutrition, yield and nutrient export in common bean under zinc fertilization in no-till system. Ciênc. Agrotec..

[72] Rashed H.O., Jahangir M.M.R., Hashem M.A., Ferdous J., Kader M.A., Solaiman Z.M., Hoque T.S. (2023). Critical Limit of Manganese for Soybean in Soils of Three Agro-Ecological Zones of Bangladesh. Sustainability.

[73] Wege S. (2022). Manganese management in plants: Golgi transporter determines manganese allocation and cell wall composition. Plant Physiol..

[74] Jin X., Ackah M., Wang L., Amoako F.K., Shi Y., Essoh L.G., Li J., Zhang Q., Li H., Zhao W. (2023). Magnesium Nutrient Application Induces Metabolomics and Physiological Responses in Mulberry (*Morus alba*) Plants. Int. J. Mol. Sci..

